# Collection and reporting of waste data to support waste management policies

**DOI:** 10.1038/s44296-025-00092-6

**Published:** 2026-02-21

**Authors:** Chunbo Zhang, Léonel Tchadjié Noumbissié, Jishuo Zhang, Chi Zhang, Stijn van Ewijk, Julia A. Stegemann

**Affiliations:** Department of Civil, Environmental and Geomatic Engineering, University College London, London, UK

**Keywords:** Engineering, Environmental sciences, Environmental social sciences, Mathematics and computing

## Abstract

Policies for waste management, including target setting, infrastructure planning and circular economy interventions, crucially depend on the availability and quality of waste data. This paper evaluates the Waste Data Interrogator for England (WDI), a key database compiling reports from permitted waste facilities managing controlled wastes and compares it with six other European databases. Its usefulness for policy-making was evaluated across five key aspects: classification effectiveness, information comprehensiveness, suitability for spatial analytics, clarity and user-friendliness and reliability. The analysis focused on construction, demolition and excavation waste, which is the largest waste stream in the database and in many regions globally, and a common policy priority. Our analysis of the WDI in a European context provides internationally relevant insights and identifies areas for improvement. Recommendations include refining classification to inform recovery, broadening statistical coverage, strengthening spatial detail, enhancing metadata and promoting digitalisation, all of which support better evidence-based waste management policy-making.

## Introduction

The modern world is experiencing an exponential surge in waste generation driven by increasing affluence and rising consumption patterns. Globally, 25.4 Gt of solid and liquid waste were disposed of in 2020^1^. This amount of waste generation poses significant challenges for waste management systems worldwide, highlighting the urgent need for innovative and sustainable solutions to manage our material resources^2^.

Effective waste management can prevent waste from contaminating ecosystems and adversely affecting human health, but also conserve resources and create substantial economic value and job opportunities^3^. Waste hierarchy and circular economy are closely related concepts that promote more sustainable resource management^4^. The waste hierarchy, described in the EU Waste Framework Directive (WFD)^5^, prioritises waste management options from the most preferable, prevention, through different recovery (‘R’) practices, to the least preferable, disposal^5^. This aligns with the R-principles of the circular economy across the pre-use, use and post-use phases of materials^4^. There is increasing emphasis on the implementation of the waste hierarchy^6,7^ and circular economy principles^8^, at scales from local to global, ideally culminating in the establishment of a zero-waste and resource-efficient society.

Waste data play a critical role across three interrelated dimensions: process evaluation, planning and regulation. At the process level, detailed data enable independent assessment of operational efficiency and environmental performance across facilities. At the planning level, aggregated company and regional statistics inform evidence-based decisions by both governmental and non-governmental actors, supporting infrastructure development and policy evaluation. At the regulatory level, national statistics ensure benchmarking and compliance with national and international standards and targets. These dimensions are mutually reinforcing—process data underpin planning, while regulatory targets guide data priorities. This study focuses on the national statistics that are fundamental for understanding regional waste management systems, including identifying problematic waste streams, predicting future waste generation, and optimising waste and resource planning, including for recovery, to reduce waste and its impacts. In the EU, waste statistics, mainly provided by the Statistical Office of the European Union (Eurostat)^9,10^, are essential for shaping and monitoring compliance with waste management policies and regulations^11,12^. This includes the EU pollution reduction targets^13^, the 2030 Circular Material Use target^14^, the 2020 construction and demolition waste (CDW) recovery target^5^ and carbon peak and neutrality commitments^15^.

However, waste statistics are widely criticised for their limited ability to effectively inform policy-making^16^. This is largely due to persistent shortcomings such as vague terminology and definitions^11,17^, limited data quality^11,17–20^, incomplete scope^11,16,19,21^, poor comparability and interoperability^11,17,18,20^ and inadequate classification and granularity^11,16,19,20^. The Eurostat datasets, while among the most comprehensive sources of waste statistics in Europe, are not exempt from these challenges. Reported issues include discrepancies in data collection methods among member states^20^, quantity mismatches between generation and treatment^22^; and loss of granularity and potential misinterpretation when aggregating site-based operational data to national and subsequently to EU levels^17^, which limit the reliability of waste statistics.

This study aims to evaluate the usefulness of a representative national waste database in a European context, informing international waste management policies and promoting practices higher up the waste hierarchy. The Waste Data Interrogator for England (WDI), a waste database developed by England’s Environment Agency (EA) to track waste management activities, is selected for study because it is one of the most established databases of its kind, while its structure and scope resemble those for other national waste statistics in Europe. Although the context of this study is European, the issues investigated are relevant to waste policy in many other countries and regions, such that this work is of interest for a broader international audience. While the primary purpose of the WDI is compliance monitoring, it has also traditionally supported the UK Department of Environment, Food and Rural Affairs (Defra) and local authorities in planning new waste infrastructure and assessing progress towards statutory waste targets^23^.

To examine linkages between the abstract data and the most important practical challenges in waste management, and to illustrate our findings in an insightful way, this study focuses on the construction industry, which is one of the largest consumers of materials and producers of waste in the UK, as in many regions globally. Waste statistics published by Defra suggest that 69% of the total waste arisings in the UK in 2022 was construction, demolition and excavation (CD&E) waste^24^. While the industry achieves a high diversion rate from landfill (~87%), challenges remain in tracing the full chain of custody, with many Waste Transfer Notes incomplete and gaps in data transparency^25^. This underscores the need for more stringent reporting requirements, improved waste data capture, better reporting consistency and enhanced accountability across the supply chain to support reuse, recycling and circular resource management. Given its scale and strategic importance^26^, improving the accuracy and utility of CD&E waste data is critical for advancing national circular economy objectives and achieving the overall ‘Zero Avoidable Waste (ZAW)’ goal articulated in the *Waste and Resources Strategy for England*^27^.

Our analysis first compares the scope among the WDI, Eurostat datasets and five other national databases of EU member states—the Netherlands, Germany, Denmark, France and Spain—to establish the WDI’s representativeness. An in-depth analysis of the WDI is provided regarding its scope, structure, application in previous studies and the circumstances that result in multiple-counting of arisings. This is followed by an evaluation of the usefulness of CD&E waste statistics from the WDI for policy-making against five criteria. Practical recommendations for improving waste statistics in the collection, classification and presentation of data are then presented.

This paper also provides practical guidance to potential users of the WDI database, which does not currently exist. Given the basic similarity between the WDI and other national waste statistics, the analysis is also highly relevant to other parts of Europe, which face comparable challenges and opportunities. The relevance of our findings extends beyond Europe, as many countries—both those seeking EU membership and others—have adopted comparable frameworks^28^. The recommendations regarding improvements apply to any government seeking greater control of waste management flows, with many countries currently lacking the extensive data collection infrastructure common in Europe.

## Results

### Comparison of waste statistics and their classification systems

Assignment of wastes to different classes and categories is necessary for the efficient administration of their management^29^ and is therefore usually required by waste management regulations. The EU has required member states to submit waste generation and treatment data to Eurostat biennially since 2002, according to the Waste Statistics Regulation (WSR)^30^. The waste statistics collected by Eurostat use the European Waste Classification for Statistics (EWC-Stat)^31^, a standardised substance-oriented classification system. The waste categories in the EWC-Stat are aggregations of the origin-based waste codes in the European List of Waste (LoW)^32^.

While all EU member states provide ostensibly consistent waste statistics to Eurostat for the EWC-Stat categories, most individual countries collect more detailed national waste statistics based on the LoW codes on an annual basis. Following the WSR, the Waste (England and Wales) Regulations^33^ require regulated waste management sites to provide quarterly returns of information (referred to as ‘site returns’ in the following) about waste movements to and from their sites, by LoW codes, to the EA. Defra has annually provided online open access to the information returned by more than 6000 permitted sites since 2006, in the form of the WDI database^23^. Since Brexit, the EA has continued to align WDI reporting with the same Eurostat definitions, classifications and quality requirements, ensuring continuity and comparability with EU waste statistics.

The EWC-stat and LoW are two typical waste classification systems, structured hierarchically to allow different levels of reporting detail. At the broadest level, the EWC-Stat contains 13 main material-oriented categories (e.g. mineral wastes, or metallic wastes^31^). These are divided into 44 subcategories, which are further refined into 83 detailed waste types. At the most granular level, the classification distinguishes between hazardous and non-hazardous waste, resulting in 117 specific items. For statistical reporting purposes, Eurostat uses a set of 51 categories, which combine 30 non-hazardous and 21 hazardous waste types. This multi-level structure enables both high-level overviews and very detailed waste statistics, depending on the analytical needs.

The waste categories in the EWC-Stat are groups of the 824 codes in the LoW^32^. The LoW classifies waste into 20 chapters based on the sector of origin (first two digits)—for example, Chapter 17 covers CD&E waste. Within each chapter, subcategories are organised by the specific generating process (third and fourth digits) and the physicochemical characteristics of the waste (fifth and sixth digits), e.g. 17 01 01 for waste concrete. Hazardous wastes are marked with an asterisk (*). The LoW code can be mapped to the EWC-Stat code using an EU guidance document^34^.

In addition to waste classification by substance and origin, waste treatment in the EU is also classified using Recovery and Disposal (R&D) codes (Supplementary Table 1) in the EU, which identify specific methods and processes applied to waste recovery materials or disposal^5^. There are a total of 13 Recovery (R) codes and 15 Disposal (D) codes, each representing different treatment operations within its group.

Table 1 presents a comparison of the scope of the WDI, Eurostat and other waste databases from five other EU member states, and highlights that waste classification and treatment classification are applied across these datasets. Among the national waste statistics datasets examined, the WDI stands out as particularly comprehensive and informative, although other databases also have their distinct strengths. The Dutch database offers a Waste Hierarchy-based treatment categorisation^35^, distinguishing ‘reuse’ and ‘recycling’ from other operations. The Danish database provides a more detailed origin^36^, distinguishing between two classes—E (abbreviated from ‘Erhverv’, commercial in English), and H (abbreviated from ‘husholdning’, household in English)—within a Waste Fraction Code system^37^. It also includes Nomenclature for Economic Activities (NACE) codes to further specify the origin of waste^37^. The French and German databases are less accessible than the others. The French database compresses different types of data into a single column within an Excel file, making direct reading and extraction impossible^38^. The German database organises waste treatment data by site category in a long-table format, which complicates data filtering. Additionally, it does not include R&D codes to distinguish between recovery and disposal processes.

**Table 1:** Comparison of different waste databases in the EU and selected European countries

	EU	UK	Netherlands	Germany	Denmark	France	Spain
Developer	Eurostat	Defra	MIWM	Destatis	EPA	INERIS	MITECO
Database name	env_wasgen^9^, env_wastrt^10^	Waste Data Interrogator^23^	Unnamed Internal dataset^35^	32111-0004^39^	Waste Statistics^36^	Industrial Installations Discharging Pollutants^38^	PRTR Waste Statistics^86^
Data granularity level	National	Site return	National	National	Site return	Site return	Site return
Data access methods	Web portal data browser	Excel (raw data and user interface)	Excel (raw data)	Excel (raw data)	Excel (raw data)	Excel (raw data)	Excel (raw data)
Waste activity	Waste generation and treatment	Waste movement (domestic and international) and treatment	Waste treatment	Waste treatment, collection, transfer and import	Waste treatment, import and export	Waste treatment	Waste treatment
Language	English, German, French	English	Mix of Dutch and English	German	Danish	French	Spanish
Temporal scope (accessible to the authors)	2010–2020 (biennial)	2006–2022	2002–2020	2006–2021	2011–2020	2003–2022	2007–2022
Geographical scope	EU27 and other European countries	England	Netherlands	Germany	Denmark	France	Spain
Waste classification	Hazard class (i.e. hazardous and non-hazardous), EWC-Stat code	Hazard class, waste form, LoW code, EWC-Stat code	Hazard class, EWC-Stat code	LoW code	Hazard class, LoW code, Waste Fraction Code, NACE code	LoW code	LoW code, PRTR-Spain code
Treatment classification	Aggregated category of waste management operations based on R&D code, NACE code	Waste fate, R&D code	Waste Hierarchy-based treatment categorisation	Type of treatment plant	R&D code	R&D code	R&D code

Note: EWC-stat code: European Waste Classification for Statistics^31^, LoW code: European List of Wastes code^32^, R&D code: Recovery and disposal code^5^, NACE code: Nomenclature for Economic Activities^37^, Waste Fraction Code: A Danish waste classification system that classifies waste into two main categories—household waste and commercial waste^91^, PRTR code: Spanish Pollutant Release and Transfer Register code (‘Registro Estatal de Emissions y Fuentes Contaminantes’, PRTR-Spain)^92^, which is part of the European Pollutant Release and Transfer Register(E-PRTR)^93^, Defra: Department for Environment, Food & Rural Affairs, MIWM: Ministry of Infrastructure and Water Management, Destatis: Federal Statistical Office of Germany, EPA: Danish Environmental Protection Agency, INERIS: French National Institute for Industrial Environment and Risks, and MITECO: Spanish Ministry for the Ecological Transition and the Demographic Challenge.

Eurostat is the only source that reports both waste generation and treatment activities, while the WDI and the other databases focus on the treatment of collected waste. However, it is unclear whether the reported ‘generation’ data represent actual waste generation or are instead derived from collected waste; the reasons for this uncertainty are discussed later. Because it uses aggregated EWC-Stat codes, Eurostat does not directly report quantities for CD&E waste. The Dutch database has the same limitation. The WDI and the databases from Denmark, France and Spain provide more granular site-level waste data, whereas Eurostat and the Dutch and German databases offer aggregated national-level data^39^.

The WDI captures domestic waste movements within England by providing two datasets, for *Waste Removed* and *Waste Received* from permitted sites. The WDI and the German and Danish databases also report international waste imports and/or exports. The German database only provides total quantities of waste delivered from domestic and foreign sources without specifying locations. In contrast, the Danish database reports the countries involved in waste exports and imports, using Basel Convention Harmonized System (HS) codes^40^ alongside OECD (Organisation for Economic Co-operation and Development) waste codes^41^. These two coding systems often complement each other and are commonly used to monitor hazardous waste shipments within OECD countries. However, their application does not fundamentally differ from that of the LoW and R&D codes.

Despite differences in scope and structure, these waste statistics consistently include the key aspects of waste activity, waste classification and treatment classification. All seven sources use the LoW and/or EWC-Stat for waste classification. Waste treatment data are included in all databases, with the WDI as well as the Danish, French and Spanish databases specifically employing R&D codes to classify various recovery and disposal processes. Although the WDI is the most well-established database, its structure, coding approach and data granularity are broadly comparable to those of the other national datasets, making it not only the most accessible but also a representative example of the general waste statistics landscape.

### Database scope and structure

The temporal scope of the investigation into the WDI encompasses the years 2006–2022, as the most recent year of data available at the time of the study. Analysis focusing on the year 2021 offers a snapshot of CD&E waste management practices. The geographic scope of the WDI and this study is England, which makes up around 90–91% of the CD&E waste generated in the UK for the period 2010–2020^24^. The waste included in the WDI spans the full range of waste classes in the LoW.

**Fig. 1:**
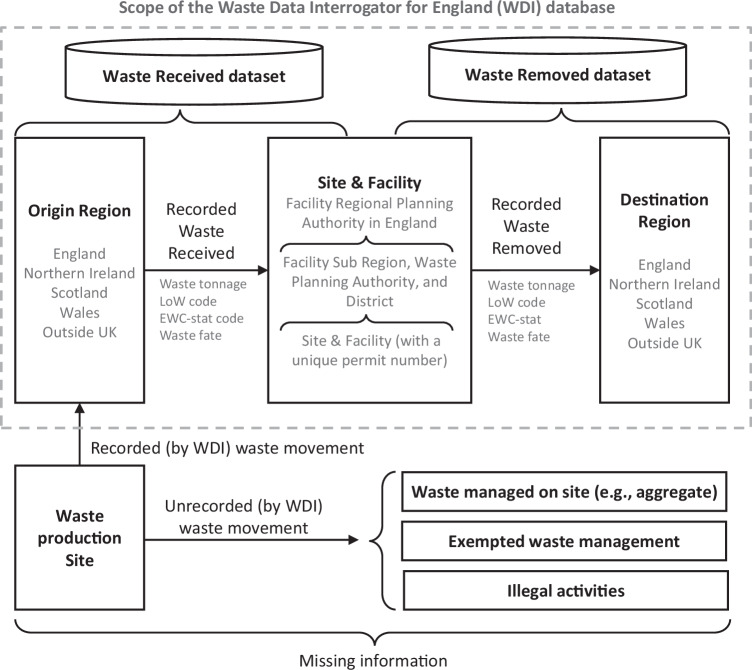
Structure and scope of the Waste Data Interrogator for England database of waste transfers to and from permitted waste management sites. Note: ‘LoW’ refers to the European List of Waste^32^.

The structure of the WDI is depicted in Fig. 1. The database is divided into two datasets: Waste Received by, and Waste Removed from, permitted waste management sites. Table 2 lists the information in each entry of both datasets, including: (i) waste classification, (ii) waste quantity, (iii) waste origin/destination region, (iv) site and facility region, (v) site and facility information and (vi) waste fate. The structure of the two datasets is essentially the same, but the Waste Received dataset reports waste flows from origin regions to specific sites, whereas the Waste Removed dataset records waste flows from specific sites to destination regions. Both ‘site’ and ‘facility’ refer to the same waste treatment location, while the term ‘facility’ is used to further subcategorise a site. Since each site is assigned a unique permit number, records in the Waste Received or Waste Removed can be linked, revealing waste transfers between sites. However, the specific treatment applied to the waste remains unclear and a single site can contain multiple facilities, waste movements between which are hidden. Both datasets report the treatment of waste in each record according to its ‘fate’, which indicates the technological processes expected to be applied at the next destination, such as incineration and long-term storage, as well as the applicable R&D code. Since waste may be treated in more than one facility, the indicated fate may not be the ultimate treatment.

**Table 2:** Comparison of Waste Received and Waste Removed datasets in the Waste Data Interrogator (WDI)

	2021 Waste Received dataset Ver. 2 & 2021 Waste Removed dataset Ver. 3	Example entries
Waste classification	Waste form	Gas; Solid
	Waste hazardousness	Hazardous
	LoW^a^ code	17 04 05 Iron and steel
	EWC-stat^b^ code	06 – Metallic wastes
Waste quantity^c^	Tonnage received/removed	42.68 tonnes
Waste origin/destination^d^ region^e^	Origin/Destination Regional Planning Authority (RPA)	East; East Midlands
	Origin/destination Waste Planning Authority (WPA)	Lincolnshire; Kent
	Origin/destination district	Lincoln; Swale
Site & facility region^e^	Facility RPA	East; East Midlands
	Facility sub region	Lincolnshire; Kent
	Facility WPA	Lincolnshire; Kent
	Facility district	Lincoln; Swale
Site & facility Info	Permit number	10338
	Permit type	A19: Metal Recycling Site (Vehicle Dismantler)
	Site name	London And Kent Metals
	Site category^f^	Combustion; Landfill
	Facility type^f^	Anaerobic digestion; Biological Treatment
	Operator	Gary Eastwood; A4 Metal Recycling Ltd
	Coordinate	Easting 591409, Northing 164462
	Postcode	ME10 3RY
Waste fate	Fate Recovery & Disposal code^g^	Incineration; Transfer (D) D01.01; R03.01.01

^a^LoW refers to the European List of Wastes^32^.

^b^EWC-Stat refers to European Waste Classification for Statistics^31^.

^c^The Waste Received dataset records received tonnages, and the Waste Removed dataset records removed tonnages.

^d^The Waste Received dataset records origins—geographical regions where waste is transferred from, while the Waste Removed dataset records destinations—geographical regions where waste is transferred to.

^e^England has nine official regions^72^.

^f^The WDI provides information on the site and facility category for each data entry. The WDI also offers explanations for each site category in its glossary.

^g^Recovery & Disposal (R&D) codes are used to indicate the waste treatment methods^5^.

The information outside the system boundary (dotted line in Fig. 1) is not provided by the WDI database. As a result, the quantity of CD&E materials estimated using Chapter 17 represents only a subset of the total arising in England. For example, whereas the generation of 73 Mt of CD&E waste in 2022^42^ can be calculated under Chapter 17 using the WDI, official statistics report a total of 132 Mt of CD&E waste^24^. Moreover, some CD&E waste arising with other wastes and CD&E waste sent to landfill from transfer and treatment stations are recorded under Chapter 19 (waste from waste management facilities…)^43^. Venkataraman et al.^44^ estimated that ~8.9 Mt of CD&E waste were landfilled under the code 19 12 12. Missing information also relates to: (i) waste directly exported or managed in situ by the producer (e.g. on-site backfilling or reuse), (ii) exempted waste management activities and (iii) illegal activities (e.g. waste trafficking and illegal land disposal)^43^, as discussed in the next paragraphs.

Firstly, information on waste handling only by producers is unavailable as the database only collects information on permitted treatment facilities. Some end-of-life materials are not classified as waste by producers, as they are not discarded in the sense described by the WFD. For example, useable timber from demolition may be separately collected for reuse by the producer or other organisations without entering waste management. Also, the EA has established 13 Quality Protocols that define End-of-Waste criteria for producing and using specific waste-derived materials^45^. For example, secondary aggregates that meet the relevant British Standard (BS) and European Norms (EN)—such as BS EN 12620: Aggregates for Concrete—are considered to have achieved end-of-waste status and are no longer subject to WFD waste management controls^46^. As a result, these secondary aggregates are excluded from waste tonnage returns and remain unreported in the WDI^47^.

Secondly, waste operations in England can be exempted from environmental permitting or reporting requirements through formal registration with the EA^48^. These exemptions apply when handling limited quantities, with specific maximum thresholds and periods defined for different waste types and operations^49^. There are currently more than 130,000 registered waste exemption holders in England, 34% of whom hold the exemption type of ‘use of waste in construction’^50^. Yet, their quantities are not reported. The thresholds are not helpful in estimating exempted quantities, as exemptions are sometimes used to disguise illegal processing of excessive quantities or materials other than those exempted; on the other hand, exemptions are free and may also be registered but never used. Use of inert rubble for farm track construction has been one of the most frequently applied exemptions. The proportion of exemptions differs for different sectors, but, for example, it was estimated that 19% of commercial and industrial waste was managed through exemptions in England in 2012^49^. It seems probable that the proportion of exempted waste has increased over time to decrease administrative burdens.

Finally, to avoid treatment and disposal fees, producers or (usually informal) waste management companies sometimes choose to illegally dump, incinerate, or landfill wastes^51^. Although waste collected from fly-tipping sites eventually becomes an entry in the WDI, illegal dumping is not recorded on a national scale. According to a waste crime survey^52^, 18% of all waste generated in England is illegally managed, ~34 Mt.

The amounts of waste reported in both WDI datasets should be equal based on the principle of conservation of mass. However, the amounts recorded in the Waste Received dataset are much larger than those in the Waste Removed dataset, for all LoW codes, partly because of the missing information noted above, i.e. received wastes are not recorded as removed from a producer, nor if they are subsequently exempted. Additionally, (i) treated wastes for further landfilling are not removed, (ii) the mass of some wastes is substantially reduced by treatment such as dewatering, sorting or incineration so less waste is removed than received and (iii) the status of some materials as waste may change after they are received—for example, if they meet End-of-Waste criteria^53^ and are reclassified as products—which means they are no longer recorded as waste when removed from the site for secondary use.

### Application of the WDI in research and multiple-counting of waste flows

A mini review was conducted on research that used the WDI, as summarised in Table 3. The reviewed studies cover scales from the company level^54^ to the whole country of England^55,56^; waste streams from particular material types, e.g. plastic^57^, to aggregated categories such as municipal solid waste^58^, CD&E waste^59^ and all waste types^60^; and sectors from construction^59^ to agriculture^57^ and fisheries^57^. Their purposes are diverse, including (i) understanding waste generation patterns; (ii) monitoring compliance and enforcement, (iii) planning and policy-making, (iv) supporting resource recovery, (v) facilitating environmental impact assessment and (vi) identifying key waste streams, such as those with the highest volume, hazardous characteristics, or significant economic value.

**Table 3:** Case studies involving the application of the Waste Data Interrogator database

Literature	Geographical scope	Target waste	Time	Dataset	Objective
^60^	Cumbria County	All waste streams	2014	Waste Received and Waste Removed	Identifying key waste streams; analysing patterns of waste arisings, management and export in Cumbria
^54^	EnviroWaste London Ltd.	All relevant waste streams managed by the company	2015	Not mentioned	Exploring how waste management companies can create a circular strategy
^55^	England	All relevant waste streams	2016	Not mentioned	Quantifying non-agricultural sources of ammonia from waste treatment
^56^	England	Microplastics	2019	Not mentioned	Assessing the impact of flooding on the release of microplastics from waste management facilities
^61^	Leicestershire	Waste streams containing nitrogen and phosphorous	2019	Waste Received	Managing nutrient flows in waste streams
^59^	West Midlands	Construction, demolition and excavation wastes	2022	Waste Received	Monitoring the production and supply of secondary aggregate
^57^	South West England	Plastic waste in agriculture and fisheries	2023	Not mentioned	Estimating the amount of plastic waste generated by agriculture and fisheries
^58^	London	Residual waste of municipal solid waste	2020	Waste Received	Evaluating treatment fractions of household residual waste

Note: the report from the Aggregate Working Party (AWP) in the West Midlands was selected as a representative case, but AWPs in other regions of England (e.g. London, East Midlands) have also mentioned the use of the WDI.

Some studies pointed out that the data collected in the WDI refers to waste movements rather than waste arisings or final treatment^58,60,61^. Originally instituted to establish a chain of custody in connection with the Duty of Care for waste^62^, reporting on waste movements can expose the economic and environmental burdens of long-distance waste transport, and help to develop strategies to reduce these^61^. However, collecting data on waste movements obstructs the assessment of waste arisings and treatment^58,60^.

Collecting statistics based on waste movements may lead to multiple-counting when estimating waste arising quantities^58,60^, as wastes removed or received from another permitted facility are counted more than once (unless they are removed under an exemption). Moreover, the WDI database is divided into two separate datasets, Waste Received and Waste Removed. While having two datasets can enable more detailed tracking, it also demands laborious integration to use both and clear reporting by analysts to avoid confusion about which dataset was used in a study^54–57^.

**Fig. 2:**
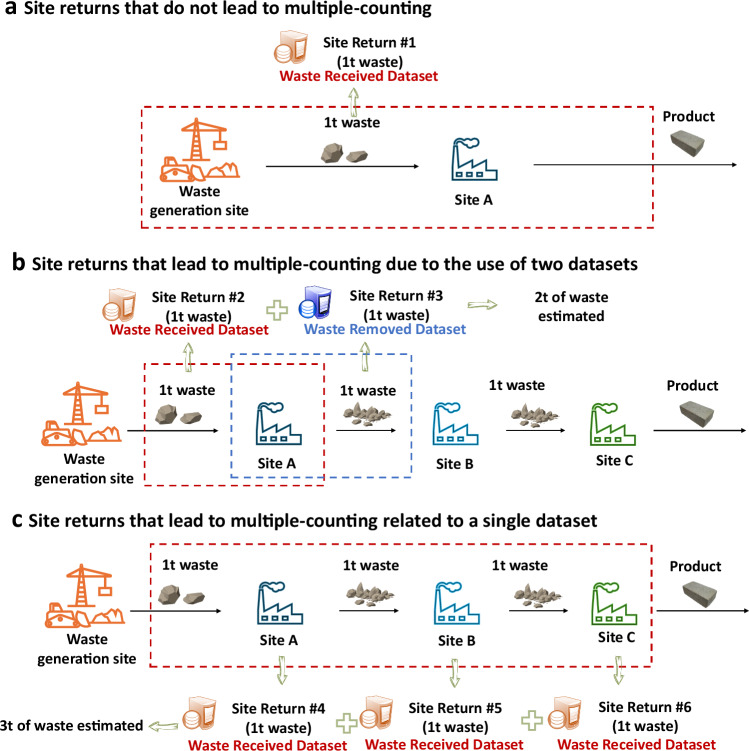
Relationship between site returns and multiple-counting. **a** Site returns that do not lead to multiple-counting. **b** Site returns that lead to multiple-counting due to the use of two datasets. **c** Site returns that lead to multiple-counting related to a single dataset.

Figure 2 illustrates how information about waste transfers returned to the EA by sites, and collected as individual records in the WDI, results in multiple-counting. Figure 2 uses three hypothetical cases to illustrate the causes of multiple-counting. Figure 2a shows that waste only undergoing one treatment process, i.e. only received by one site, does not lead to multiple-counting. Yet, waste processed at different sites sequentially leads to more than one return of information from each of these sites to the EA, resulting in multiple records in the WDI. Two types of multiple-counting exist: (i) using the Received and Removed datasets simultaneously (Fig. 2b) and (ii) using only one dataset (Fig. 2c). Figure 2b demonstrates that using the receipt and removal records of the same waste stream could result in multiple-counting. Figure 2c shows that even when working with a single dataset (the Waste Received dataset), using multiple receipt records of waste can lead to multiple-counting.

There is no definitive method to handle multiple-counting in the WDI, although attempts were made in several studies^58,60,61^. Agile Initiative^61^ states that waste flows for ‘transfer’ purposes, as opposed to treatment or disposal, could result in multiple-counting. Therefore, it attempts to eliminate multiple-counting from the Waste Received dataset by excluding entries with the same Facility Waste Planning Authority (WPA) as the Origin WPA and a site category of ‘transfer’. However, this method does not exclude transfers between different WPAs. Additionally, the term ‘transfer’ appears not only in the site categories but also in facility types and waste fates in the database and R&D codes^63^. Therefore, the exclusion of transfers based solely on site categories and WPA does not seem to be sufficient to fully eliminate multiple-counting.

Cumbria County Council^60^ attempts to exclude multiple-counted waste streams by first calculating the tonnages of waste removed from individual transfer and treatment sites in Cumbria to other sites within Cumbria using the Waste Removed dataset and then deducting these from the tonnages in the Waste Received dataset. However, this approach is also flawed, for instance, where the amount of waste removed exceeds that received, resulting in negative waste arisings. Minhas et al.^58^ found that amounts of residual municipal solid waste recorded in the WDI for London were higher than those in a local authority database, WasteDataFlow, probably due to multiple-counting. They further identified R&D codes that potentially indicate multiple-counted waste streams, but did not specify how to deal with these codes. The remaining studies^54–57,59^ did not explain whether or how the issue of multiple-counting was addressed in their use of the WDI.

### Classification should inform recovery

The diverse applications of the WDI demonstrate its strong potential to support evidence-based policy-making. C&DE waste provides a case example to assess how well existing waste statistics align with policy needs based on five criteria: (i) effectiveness of classification, (ii) comprehensiveness of waste information, (iii) suitability for spatial analysis, (iv) clarity and user-friendliness and (v) data reliability.

**Fig. 3:**
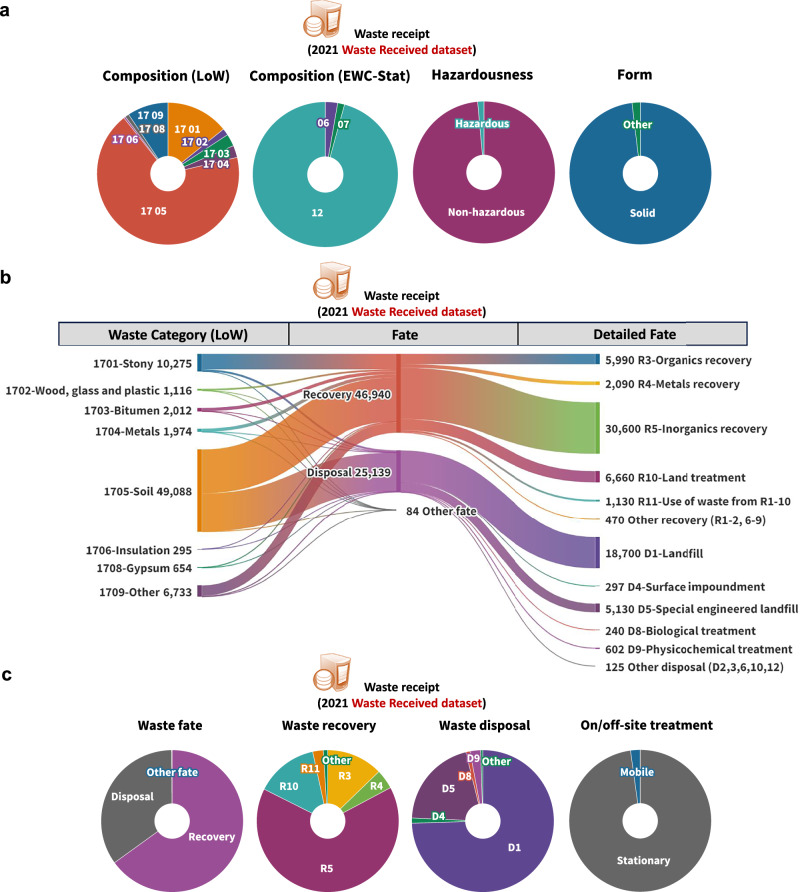
Breakdown of 72 Mt of construction, demolition, and excavation (CD&E) waste received in England (by mass) based on the 2021 Waste Received dataset. **a** Classification of CD&E waste received; LoW: European List of Waste (LoW)^32^, EWC-stat: European Waste Classification for Statistics^31^. **b** Final treatment of CD&E waste (unit: kilotonne); Recovery and Disposal (R&D)^5^ codes are used to classify the treatment (see Supplementary Table 1). **c** Classification of CD&E waste treatment.

Whereas the WDI applies the most widely accepted systems for classification, it fails to record waste in a manner that directly informs decision-makers about the potential for reuse and recovery. Both the origin-based LoW and, since 2019, the substance-oriented EWC-Stat classification codes are recorded in the WDI, along with a description of the physical nature (form) of the waste and its hazardousness. Figure 3a shows the breakdown of CD&E waste (Chapter 17 in the LoW) received in England in 2021 based on the LoW codes, EWC-Stat codes, its hazardousness and its form of physical nature. The historical trends for received waste categories and waste receipt regions in England from 2006 to 2022 are provided in Supplementary Fig. 1. Only a small fraction of CD&E waste is hazardous (presumably due to the presence of asbestos, paints and oils) or in a form other than solid. The EWC-Stat and Hazardousness classifications are largely redundant, as the relevant information is already embedded within the LoW codes. Similarly, form-based classification adds limited value for CD&E waste, as the vast majority is solid. In fact, specifying form can be misleading, as it is often unclear whether gaseous or liquid substances refer to the waste itself or to emissions generated during its treatment.

R&D codes are used to indicate the treatment of CD&E waste. Figure 3b presents the final treatment of each CD&E waste stream in England in terms of recovery, disposal and other fate (unknown or not specified) based on the R&D codes. A full-scope Sankey diagram tracing waste flows from their origin to final treatment is provided in Supplementary Fig. 2. Figure 3c further breaks down the fractions of treatment routes in 2021. Supplementary Fig. 3 shows the trend for CD&E waste treatment for the period 2017–2022. The R&D codes can be used to calculate the recovery rates of CD&E waste by LoW code (i.e. for each waste stream in each sector), a crucial indicator for assessing the performance of the current waste management system. An example of recovery rates calculated for individual CD&E waste streams is provided in Supplementary Fig. 4. Since the WDI is constructed using site-level returns, which are subsequently aggregated to the country scale, CD&E waste recovery rates can also be calculated at multiple spatial levels—site, district, region, and country (Supplementary Fig. 5). The WDI also records whether waste is processed at a mobile or stationary facility (Fig. 3c), which can inform strategies to enhance on-site recycling. The quantity of CD&E waste received at each site or facility can be calculated (Supplementary Fig. 6), but this information does not yield substantial new insights into waste management, as the final fate is more important than the facility used. Figure 3 and other figures in this study illustrate the information and patterns obtainable from the WDI, but the presented quantities should be interpreted with caution due to inherent uncertainties and the incompleteness of the WDI datasets.

Figure 3 illustrates that the WDI offers a wealth of information, but not much of it can be used to gauge the usefulness of the waste. It is a fundamental flaw of both the waste and treatment classification systems—origin-based LoW, substance-based EWC-Stat and R&D codes—used by the WDI that they do not categorise wastes in a way that supports their recovery at the highest value, i.e. by providing the information needed for their reuse or recycling. The classification of waste according to composition (e.g. metals), hazardousness and form (e.g. solid) provides a starting point for assessing reuse and recovery pathways, but critical aspects such as quality and contamination are not included, without which it is impossible to tell whether the waste may be used.

A key element in improving the usefulness of waste statistics lies in how the origin of waste is identified and classified. Identification of the industries and processes of origin for waste, as in the LoW, is perhaps the simplest basis for implementing the chain-of-custody based waste data collection, such as the WDI, since it does not require any detailed technical knowledge or judgement by operators or carriers. Coverage of additional information about waste generation activities (e.g. construction, demolition, or renovation, for CD&E waste) and sectors (e.g. residential, service, or infrastructure) would enhance understanding of waste generation patterns and facilitate the development of sector- and activity-specific interventions for the recovery of materials at higher value. This approach would also support industrial symbiosis, in which one organisation’s waste becomes another’s raw material in an interconnected industrial network^64^. Including such additional information would require a new coding system more complex than the Waste Fraction Code^36^ and NACE codes^37^, which have already been demonstrated by the Danish waste database^36^, perhaps with connection to more detailed (and possibly expanded) PRODCOM codes used by governments to monitor domestic production and trade^65^.

The use of more detailed R&D codes is a further requirement to support recovery, since the current system lacks detail about the technological routes used. It is currently not apparent whether a waste is reused, or downcycled or upcycled, in an open or closed loop.

A classification system related to the concept of *Use Potential*^66^ would enable a shift from merely tracking waste quantities to identifying potential recovery routes and markets. Whilst waste *Use Potential* is highly contextual, i.e. depending on technological, economic, social, environmental and regulatory factors, it also depends on waste characteristics that could be better captured by classification, e.g. pertaining to quality and contamination. The substance-oriented EWC-Stat codes are an incomplete step in this direction, and could be further developed to capture waste composition and properties. By introducing waste *Use Potential*, statistics could also help monitor the gap between theoretical recovery potential and actual recovery rates, thereby identifying inefficiencies and opportunities within the waste management chain. This would support policymakers and stakeholders in designing targeted interventions to enhance resource recovery and reduce environmental impact. To support this shift, sector-specific guidance—such as that found in the Best Available Techniques Reference Documents (BREFs)^67^, used in the permitting of facilities under the Industrial Emissions Directive^68^—could be adapted to incorporate recovery-focused classification principles.

### Scope should include generation

The WDI reports waste movements for waste treatment (both intermediate and final treatments) rather than waste generation, which therefore constitutes a gap in system coverage. Gathering waste generation data from waste producers rather than waste movement data from waste management sites would provide direct figures for waste arisings. This would enable a more accurate assessment of the balance between waste generation and waste management activities, e.g. for the development of policy interventions and monitoring of waste management targets. It would also avoid the problem of multiple-counting. In addition, providing waste generation data would enable monitoring of the success of waste prevention efforts (e.g. direct on-site reuse), which is currently absent from the investigated databases.

The WDI waste received (Supplementary Fig. 1) can, to some degree, reflect the trend of waste generation. Yet, records of direct recovery and reuse, waste managed under exemptions and illegal disposal activities are needed to understand total CD&E waste generation. Among the databases investigated, only Eurostat reports waste generation. However, the waste quantities reported in the Waste Generation and Waste Treatment datasets in Eurostat do not match. This is due to differences in statistical scope—for example, the Waste Treatment dataset includes only final treatment, where mass may be lost during pre-treatment activities and it excludes exports while including imports of waste^22^.

Reporting on waste generation involves waste producers rather than facility operators, which represents a significant departure from current waste statistics practices. One major challenge is that waste producers would need to install weighbridges or other measurement systems typically found only at waste treatment facilities, which adds logistical complexity and cost. Companies may also fear that sharing detailed waste data could expose sensitive business information and undermine their market position^69,70^. Overcoming these challenges would require not only technical solutions but also building trust and incentives for producers to participate in more transparent waste reporting systems.

Still, waste movement matters: strategically transferring processed waste to another site for temporary storage and later recovery is a useful means to enhance circularity, particularly for bulky waste like soil and concrete^71^. Ideally, the data in the WDI would capture waste generation, waste movement and the final destination of waste. This may be possible by assigning a reference number to each flow, which would follow it through all transfers regardless of any changes in mass due to treatment, automatically linking the current independent waste returns from different operators. Upon waste separation or aggregation, sub- or aggregated flows may be assigned reference numbers that incorporate the original reference(s). Blockchain (distributed ledger) technology could enhance this tracking system by providing an immutable, transparent and transparent ledger that securely records each of multiple transactions along the waste life cycle^69^. Moreover, blockchain’s decentralised access model would facilitate real-time coordination among stakeholders while protecting sensitive commercial information through permissioned access.

### Planning policy requires more spatial data

**Fig. 4:**
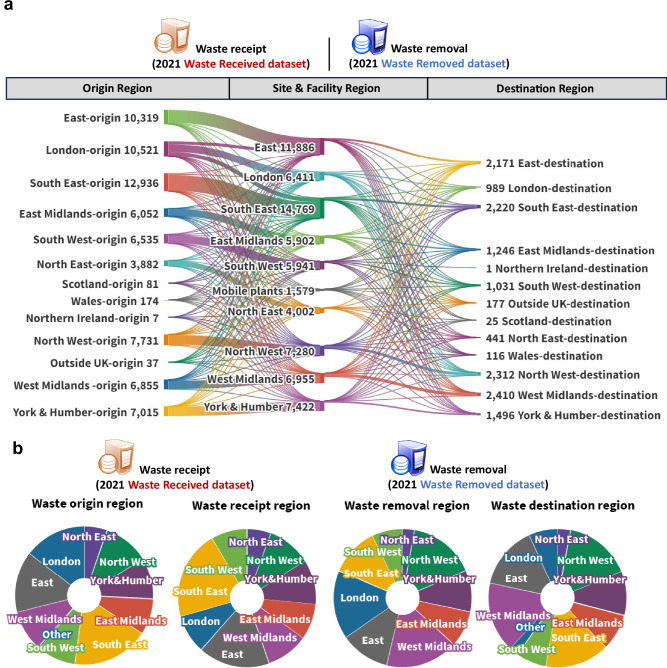
Breakdown of construction, demolition, and excavation (CD&E) waste movement (72 Mt received and 15 Mt removed) in England in 2021. **a** CD&E waste received and removed in each region (unit: kilotonne). **b** Mass fractions of CD&E waste received and removed in each region.

The WDI centres on the nine regions of England^72^, and it also reports waste movement between England and other UK nations (i.e. Wales, Scotland, and Northern Ireland), and other countries outside the UK. Figure 4 shows the potential for regional analysis. Figure 4a shows final flows for receipt and removal of CD&E waste in England in 2021 (noting that multiple-counted flows were excluded in this analysis to maintain consistency with the method in this study, and that waste received and removed do not balance, as discussed above). Figure 4b presents the mass fraction of waste movements in each region. Most waste was transported within its region of origin, with negligible amounts received from or removed to other countries.

**Fig. 5:**
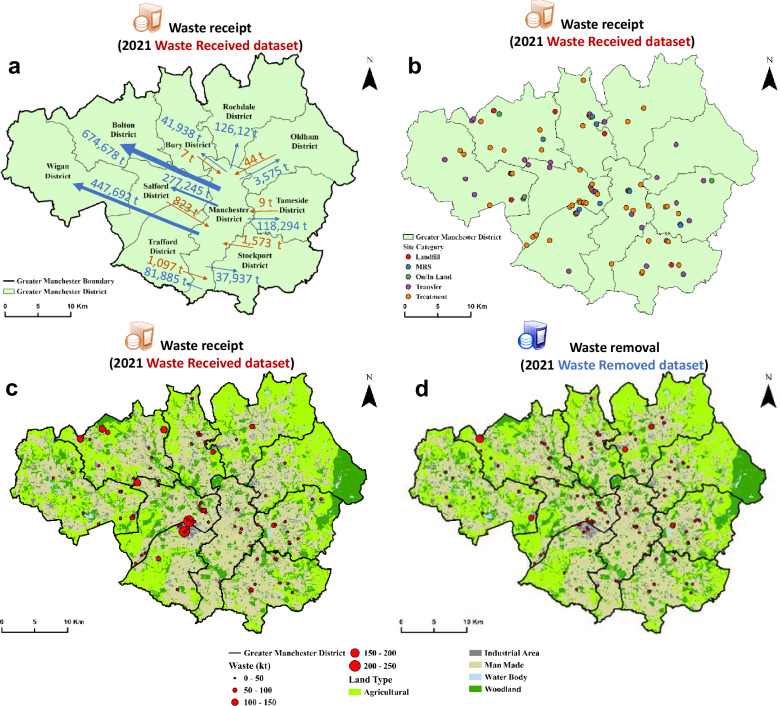
Spatial analysis of construction, demolition and excavation (CD&E) waste (2.5 Mt receipt and 1.0 Mt removal) management in Greater Manchester in 2021. **a** waste movement between central Manchester and other districts; **b** site distribution and receipt of hazardous and non-hazardous waste, **c** spatial distribution of waste receipt at sites and **d** spatial distribution of waste removal from sites.

The county of Greater Manchester in Northwest England was selected to demonstrate more detailed spatial analysis of WDI information from 2021, based on the Cartesian coordinates provided in the WDI. Figure 5a illustrates that most CD&E waste movements originated in central Manchester and were transferred to the nine other metropolitan boroughs. Figure 5b depicts the distribution of waste sites among all ten districts of Greater Manchester, with the majority of CD&E waste sites located in the centre and northwest. Figure 5c, d show the distribution of sites and the amount of waste received and removed within Greater Manchester. This indicates that the WDI is able, to some extent, to reveal domestic movements.

Figures 4 and 5 show that the WDI captures regional waste flows and their treatments, allowing policymakers to tailor recovery goals specific to local contexts. The provision of facility coordinates in the database also has the potential to support optimisation of waste transport distances, for the reduction of carbon emissions. However, the WDI cannot help to optimise collection transport from producers, since these are not recorded. In addition, the absence of data about site-level treatment capacities restricts the ability to allocate waste efficiently across facilities.

Although the WDI reports international imports and exports within England, it lacks information on the origin and destination countries that would improve understanding of the international waste management network. Databases like Comtrade^73^, developed by the United Nations, provide international trade statistics using HS codes, which classify goods, including various waste and scrap materials. Compared to Comtrade, the WDI offers more detailed waste import and export data using LoW codes.

Overall, the WDI captures spatial aspects of waste movement, but key information for waste management planning is missing. Inclusion of precise coordinates for all transfer points and detailed transport route data in the WDI, including waste producers and destinations outside England, as well as site treatment capacities, would enable policymakers to integrate carbon accounting into waste management planning.

### Enhancing metadata for clarity and user-friendliness

Clarity refers to the information environment of the data. This includes whether the data come with relevant metadata, i.e. ‘data about data’ that describes the content, quality, structure, intended use and usage limitations of the dataset^74^. User-friendliness refers to the ease of access, navigation and interpretation of the database, which enables users to efficiently retrieve, understand and use the stored information. For both clarity and user-friendliness, it is a major issue that the WDI consists of two separate datasets that are complicated to connect, particularly without official instructions on their use, e.g. to avoid multiple-counting. Moreover, the current glossary fails to clarify key terms. In particular, the lack of distinction between the point of waste generation and the point of ‘origin*’* can lead to misinterpretation. Also, ‘transfer (D)’ and ‘transfer (R)’, indicating transfer for disposal and recovery, respectively, need further explanation. To our knowledge, the present article is the first document that incorporates extensive guidance (i.e. metadata) on the WDI.

To strengthen metadata, an improved waste tracking system should incorporate explicit user instructions and contextual guidance to avoid ambiguity and enable detailed analysis, as well as clearer definitions and a glossary list of key terms such as transfer, fate and origin. More detailed R&D codes, and a clear explanation of how they should be used could prevent misunderstanding of treatment classifications and improper attribution of fates.

### Ensuring reliability with digitalisation

Reliability refers to (i) the completeness of data collection and (ii) the extent to which the data collected reflects the actual waste quantities, types and management practices without significant errors or misreporting. For the WDI, incomplete data result from factors such as direct management by the producer, exemptions and illegal activities, which lead to unrecorded waste generation and movements. These issues are common to waste statistics in other countries that report waste collection for treatment rather than actual generation. The aforementioned improvements—such as adopting more robust classification systems and extending the scope of reporting—could, to some extent, enhance data completeness by capturing a fuller picture of waste flows. Yet, achieving comprehensive completeness will require a more systemic approach.

Digitalisation has established a new norm for the systematisation of knowledge derived from physical phenomena^75^ and is proactively influencing every aspect of our society^76^. Defra aims to introduce a Mandatory Digital Waste Tracking (MDWT) service across the UK by October 2026^77^. The MDWT will digitise and integrate currently fragmented and predominantly paper-based records into a harmonised tracking system, to enhance the timeliness of waste data gathering and monitor the quantity and types of waste produced and their final destinations. One of the key features of the MDWT system will be the introduction of a unique identifier that links the waste producer, carrier and the first receiving site^78^, which is consistent with the reference number proposed in this study. However, a new identifier will be issued when the waste leaves the first site and is received by a second site^78^, and it is not yet clear whether a mechanism will exist to connect the old and new identifiers across the chain. Beyond identifiers, the system also aims to capture more comprehensive metadata, including details of the waste producer, specific waste characteristics (e.g. chemical composition and containment), destination details, Standard Industrial Classification codes (a four-digit number used to classify businesses by their primary economic activity), treatment details (including the final fate of the waste) and information on any end-of-waste products or materials generated, along with their subsequent destinations^78^.

The MDWT system can substantially enhance the completeness of information captured for the WDI, to enable greater visibility of producer waste management practices, capture details of exemptions and deter waste crimes ranging from fly-tipping and the operation of illegal waste sites to unlawful waste exports^77^.

Waste databases may also suffer from errors or misreporting. The WDI is data-intensive; the 2021 Waste Received dataset alone has over 40,000 entry rows under 27 columns of data headers. Furthermore, multiple actors, e.g. waste producers, waste carriers, site operators and database developers, are involved in data collection, reporting and recording, with many opportunities to introduce errors. The reliability of the database can only be assessed by identifying implausible entries, i.e. values that are logically inconsistent or violate known physical laws or constraints. This was tested by investigating (i) comparing quantities of waste removed and received, with the former logically having to be lower than the latter, as explained earlier and (ii) improbable fates.

Figure 5c, d reveal some sites that received no waste but from which waste was removed. Calculation of the net waste receipt (waste received minus waste removed) for all 151 sites in Greater Manchester in 2021 reveals that waste removed exceeded waste received in 85 (56%) of these (see Supplementary Fig. 7a). The average net waste receipt is ~10 kt, with a range of between 58 and 401 kt. This is consistent with short-term imbalances in movements at the beginning and end of the year, which might be expected to result in an excess of removals over receipts in about half of the sites. An excess of receipts over removals in the other half of the sites is likely, but more difficult to discern given other factors.

Concrete and metals have the highest share of improbable fates, ~11% and 4%, respectively (Supplementary Fig. 7b). The shares for other waste streams are all less than 2%. England received 3.2 Mt of waste concrete (LoW code 17 01 01) in 2021, of which 0.1% was sent for energy recovery and 5.6% was recovered through recycling/reclamation of organic substances. These fates are inconsistent with the inorganic, incombustible nature of concrete. They may result either from organic or calorific residues incorrectly recorded as concrete, or from misreporting of their management. For instance, wastes such as paper/cardboard, landscaping waste and textile-based materials (e.g. carpets) are not typically generated as primary materials in CDW; they are usually removed before demolition, and managed under Chapter 15 ‘Waste Packaging’ and Chapter 20 ‘Municipal Wastes’ LoW codes. However, residues of these materials may contaminate CDW. In practice, concrete containing minor fractions of other materials may not be categorised as mixed CDW (17 09 04), which results in reporting of ‘recycling/reclamation of organic substances (R3)’ and ‘incineration of concrete (D10)’. In addition, misclassification may also occur due to the structure of the Waste Removed dataset, where each record must specify a fate even though the site removing the waste may not be aware of the treatment ultimately applied by the receiving facility. Some implausible fates may result simply from operator errors.

The MDWT system can further improve the quality of waste data by minimising discrepancies and reducing errors associated with manual data entry in the WDI. By digitally recording the full chain of custody, it enables rapid cross-checking of mass balances between waste received and removed. In addition, automated validation mechanisms can be introduced to flag and prevent inappropriate or inconsistent waste fate reporting. Introducing temporal alignment protocols and requiring explanations for large net negative receipts could reduce artefacts caused by reporting cut-offs. Therefore, the MDWT system would greatly enhance the reliability of the waste data in terms of completeness and quality. While the technical specifications of the new system have not yet been released, its introduction in the UK has the potential to be a game-changer for waste statistics.

### Overall evaluation of policy relevance

Table 4 lists typical waste policies with quantifiable targets and considers the relevance of the WDI to those waste management policies, using examples from the EU. It distinguishes between mass-based policies and impact-oriented policies. The relevance of the WDI to the development and implementation of each policy was evaluated as ‘weak’ or ‘strong’ the main issues or opportunities offered by the database are briefly summarised.

**Table 4:** Relevance of the Waste Data Interrogator (WDI) database to waste management policies

Waste policy category		Example of quantifiable target	WDI Relevance
Waste mass-based policy	Prevention/reduction of waste production	England: Eliminating avoidable waste of all kinds in construction by 2050 (e.g. by designing out waste)^79^ EU: Significantly reducing total waste generation and residual municipal waste by 50 mass % by 2030^13^	**Level:** Weak **Remark:** Current waste classification in the WDI does not directly capture the quantities of wastes generated, nor identify the avoidability of waste, nor the potential to reduce waste through design or reuse/recover it.
	Illegal waste transport	EU: Preventing illegal shipments of waste occurring within the EU, as well as from the EU to third countries^94^	**Level:** Weak **Remark:** The WDI does not capture data on international shipments, including illegal ones; therefore, it cannot reliably reflect illegal waste transport activities.
	Waste recovery	EU: Realising recovery of non-hazardous construction and demolition waste with a minimum of 70 mass % by 2020^5^	**Level:** Strong **Remark:** The R&D codes included in the WDI indicate the share of waste recovered.
	High-value-added waste management	EU: Promoting the preparation for re-use and the recycling of municipal waste to a minimum of 55%, 60% and 65% by mass by 2025, 2030 and 2035, respectively^5^	**Level:** Weak **Remark:** Although the R&D codes distinguish between recovery and disposal, they do not identify specific recovery routes
	Restriction on waste disposal	Scotland: Prohibiting the landfill of biodegradable municipal waste going to landfill by 2025^81^ EU: Reducing the amount of municipal waste for landfilling to 10 mass % or less of the total municipal waste generated by 2035^80^	**Level:** Strong **Remark:** The R&D codes provide information on the share of waste disposal.
	Raw material displacement	EU: Increasing the circular material use rate to 22.4% by 2030^14^	**Level:** Weak **Remark:** Although the quantity of produced secondary materials could be estimated, the WDI lacks data on where and at what quality materials are recovered, posing a significant challenge for using the WDI to fully support this target.
Waste impact-based policy	Decarbonisation from waste management	EU: Reducing 68% of greenhouse gas emissions from the waste sector by 2050 compared to 1990 levels^95^	**Level:** Weak **Remark:** Data on gas emissions are not provided and cannot be directly estimated due to the vagueness of waste treatment routes
	Decarbonisation from transport (including waste transport)	EU: Reducing 90% greenhouse gas emissions in transport by 2050^96^	**Level:** Weak **Remark:** it lacks detailed information on transport modes and distances for transport-related emission estimate.
	Reduction of pollution from waste	EU: Reducing the health impacts (premature deaths) of air pollution by more than 55% by 2030^13^ EU: Reducing waste plastic at sea by 50% and microplastics released into the environment by 30%^13^	**Level:** Weak **Remark:** Data on pollution from waste is not provided nor can be directly estimated

Since the WDI is based on waste mass, it is more relevant to mass-based waste policies, such as the ZAW goal^79^ and also others for policies that aim to boost recovery^5^ and reducing landfill^80,81^, rather than impact-based waste policies. However, even for mass-based policies, the WDI falls short by omitting some wastes from tracking, which is unhelpful for policy-making, as it loses valuable sources of information regarding potentially important resource streams. Waste prevention at the design stage is challenging to quantify and capture, and the WDI is somewhat inevitably limited in its ability to support and monitor waste prevention and reduction. However, the WDI does not even provide direct information on waste generation, which is key information for monitoring of waste reduction and waste trafficking.

The importance of good waste management for mitigating climate change has been increasingly addressed by studies^26,82–84^. The usefulness of the WDI for facilitating carbon reduction is limited by the R&D code system, which fails to indicate specific treatment processes or the production of secondary raw materials. This hinders upgrading of waste management practices in line with the waste hierarchy, as well as substitution of primary raw materials. The WDI cannot support waste transport emission reduction either, as the transport modes and distances are missing from the database. Additionally, the WDI cannot be used to access pollution impacts, as it lacks both direct measurements and proxy indicators of waste-related emissions.

Incorporation of the improvements to waste statistics outlined in the preceding sections in the new MDWT, with digitalisation as a key enabler, could significantly strengthen their policy relevance across nearly all aspects of waste governance. By collecting both producer-side waste generation data and carrier transport data, with the use of unique identifiers, the system will enable more effective monitoring of waste reduction, illegal transport surveillance and transport-related emissions accounting. With detailed information on waste treatment processes and the distribution of secondary products, policymakers and industries will be able to better understand and support the transition to advanced waste management practices and raw material substitution in line with circular economy principles.

Overall, this analysis highlights the urgent need to move beyond fragmented, compliance-oriented databases towards integrated, digitalised and harmonised waste tracking systems. The MDWT provides a major step in this direction, and if effectively implemented, it could become a model for international adoption. In the long term, such systems will not only support compliance and enforcement but also enable evidence-based policy-making, accelerate progress towards circular economy targets and strengthen global efforts to decarbonise and minimise waste.

## Discussion

The preceding sections evaluated the usefulness of the WDI in relation to policy-making generally, noting shortcomings related to vague classifications that hinder material recovery, limited ability to reveal waste generation patterns, insufficient support for spatial analysis, lack of clarity and guidance for users, and persistent issues with data completeness and quality. To address these gaps, several improvements are proposed for collection of waste data in the WDI, and similar databases, including the new MDWT. First, the waste generation classification should be refined to better capture waste sources and promote industrial symbiosis, by incorporating information on the *Use Potential* of waste. Second, the database scope should be expanded to include waste generation data. Third, additional spatial data—such as precise facility coordinates and treatment capacities—should be integrated to enable high-resolution geomatic analyses. Fourth, metadata should be improved through explicit user instructions, contextual guidance and consistent terminology. Finally, waste traceability and data reliability can be strengthened through digitalisation.

While these recommendations could greatly improve the usefulness and policy relevance of the WDI, they may also increase administrative complexity and financial investment requirements for both regulators and operators. This is also the reason why the OECD includes cost-efficiency as one criterion in its statistical quality framework^85^. For example, improving the treatment classification by including waste *Use Potential* requires a waste quality assessment system that evaluates material properties, which could considerably complicate waste reporting. While the MDWT makes progress towards digitalisation, expanding the data scope and introducing technologies such as blockchain will require significant technical infrastructure, data-sharing agreements and coordination among multiple agencies. Smaller waste operators, particularly those handling niche or local waste streams, may lack the capacity to comply with more demanding data-reporting requirements. Therefore, any enhancement to the WDI should consider scalability and proportionality, ensuring that improvements in data accuracy and transparency do not unintentionally discourage reporting or participation.

Moreover, implementing finer spatial resolution and facility-level detail could raise confidentiality and data protection concerns, especially when private-sector data are involved. Balancing transparency with privacy protection will be critical to maintain trust among stakeholders. Harmonisation with existing reporting obligations under EU directives and national regulations should also be prioritised to avoid duplication and conflicting data flows.

In parallel, the effectiveness of proposed improvements depends on building institutional capacity for data management, analysis and interpretation. Even the most comprehensive datasets are of limited value without trained personnel and analytical tools to translate raw data into actionable insights for policy and planning. Hence, investment in human and institutional resources—through training, inter-agency coordination and the development of open analytical platforms—should accompany any technical enhancement of the database.

Finally, improving waste statistics should not be viewed as a purely technical task but as part of a broader governance effort to strengthen evidence-based policy-making. Better data can facilitate feedback loops between operational performance and strategic planning, enable cross-sectoral coordination and enhance accountability in achieving circular economy and carbon reduction targets. However, achieving these benefits will require a long-term vision, stakeholder collaboration and sustained policy commitment to data transparency and standardisation.

In summary, improving collection of waste statistics is not only about refining data structures but about redefining how waste information supports sustainable resource management and policy-making. The proposed measures collectively aim to bridge the gap between operational data and policy needs. While their implementation requires careful balancing of data quality, feasibility and administrative capacity, they represent an essential step towards more transparent, interoperable and policy-relevant waste statistics. Ultimately, a more robust and adaptive database system would empower both governmental and non-governmental actors to better monitor progress towards circular economy and decarbonisation goals, ensuring that waste data becomes an active driver of systemic transformation rather than a passive record of past performance.

## Methods

### Analytical framework

**Fig. 6:**
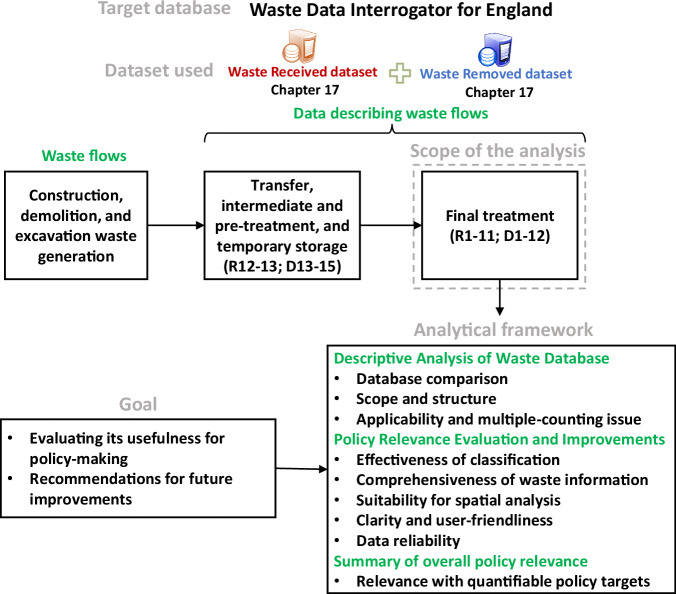
Methodological diagram of this study.

This study had two goals: (i) to evaluate the usefulness of the WDI as a representative example, and (ii) to make recommendations for improving waste statistics to better inform policy-making (Fig. 6).

This study primarily relied on the Waste Received dataset of the WDI, which provides a more complete estimate of waste arisings and management^60^. The Waste Removed dataset was also used as a complement, offering spatial information on waste movements. CD&E waste was selected as a case to illustrate the extent to which the WDI can support policy-making. CD&E waste entries were identified by screening 6-digit waste codes in Chapter 17 of the LoW, titled ‘Construction and Demolition Wastes (including Excavated Soil from Contaminated Sites)’ (see Supplementary Fig. 4 for the full list).

R&D codes identified by Minhas et al.^58^ as potentially associated with multiple-counted flows, i.e. R12, R13, D13, D14 and D15 (noting that Minhas et al.^58^ argue that D8 could also lead to multiple counting, although this was not considered within the scope of this analysis), which are related to intermediate activities prior to final treatment (Supplementary Table 1), were excluded from analysis of both datasets. This approach ensured that only quantities undergoing final treatment (i.e. final fate), were included, but had two limitations: (i) it remains uncertain whether these codes actually result in multiple counting, and (ii) mass losses during treatment processes such as dewatering, sorting, or incineration are not captured, potentially leading to underestimation of total waste generation. The analytical framework consisted of two parts (Fig. 6): (i) descriptive analysis of the WDI, and (ii) evaluation of its policy relevance and recommendations for improvement.

### Descriptive analysis

The descriptive analysis consists of three components: (i) database comparison, (ii) scope and structure analysis and (iii) identification of applicability and multiple-counting issues. Comparing different waste databases enables the identification of strengths, limitations and methodological differences in data collection, classification and reporting. This study selected seven databases for comparison, limited to those known within our project team: Eurostat^9,10^, representing EU-level waste statistics; the WDI^23^ and national waste statistics from the Netherlands^35^, Germany^39^, Denmark^36^, France^38^ and Spain^86^, collectively representing Western and Southern Europe. In 2017, Deloitte evaluated the quality of official CD&E statistics from EU member states in 2012 as good, modest, or poor^20^. The UK is considered to provide data of ‘modest’ quality, with available data from three countries rated as having good-level CD&E statistics, the Netherlands, Germany and Denmark; alongside two other countries with modest-level data, France and Spain; as well as Eurostat. The WDI was selected as the target case for in-depth analysis because its high level of informativeness enables identification of potential issues, while its representativeness ensures that the findings and limitations reflect common challenges shared by other waste statistics systems.

Covering all waste databases across EU member states was not feasible due to differences in administrative conventions, language barriers and data accessibility. For example, some member states treat data as confidential; many countries publish waste statistics only in their national language, complicating data retrieval and analysis. Furthermore, multiple databases may coexist within a single country—such as England, which maintains both the WDI and WasteDataFlow^87^—creating additional structural complexity. Consequently, this study acknowledges that examining five national databases cannot fully capture the diversity of waste statistics collection practices across Europe.

A detailed analysis of the WDI was then conducted, focusing on its scope and structure. The scope assessment examined temporal and geographic coverage as well as inclusion of waste categories. Building on this, the structural analysis investigates the completeness of waste information, potential omissions and interrelations between the Waste Received and Waste Removed datasets.

Finally, a mini-review of previous research using the WDI was carried out, with special attention to how those studies addressed potential multiple-counting issues and their adopted mitigation strategies.

### Evaluation of policy relevance

A qualitative evaluation of the WDI’s policy relevance was conducted based on insights from the descriptive analysis. Various frameworks exist for evaluating the overall quality of statistical data, including those developed by the OECD^85^, Eurostat^74^, Statistics Canada^88^, Statistics Sweden^89^ and the UK Statistics Authority^90^. These frameworks consider criteria such as relevance, accuracy, reliability, timeliness, punctuality, accessibility, availability, clarity, comparability, coherence, interpretability, content, integrity, methodological soundness, serviceability, quality, credibility, among others. Supplementary Table 2 summarises these frameworks and their respective definitions of each criterion.

However, empirical research applying these criteria to waste databases remains scarce, exemplified by the simplified qualitative scale (‘Good’, ‘Modest’, or ‘Poor’) of the Deloitte study^20^. Rather than conducting a systematic indicator-based evaluation, this study provides a critical assessment of the WDI and proposes targeted recommendations. Specifically, five aspects were adopted based on those frameworks, selected for their high relevance to waste management (as listed in Classification Should Inform Recovery). The correspondence between these five aspects and the criteria of established frameworks is detailed in Supplementary Table 3.

Based on this evaluation, recommendations are proposed to improve the WDI and enhance its policy relevance. An extended discussion is performed to illustrate how these improvements could strengthen the WDI’s ability to support evidence-based polic-ymaking by assessing its current and potential relevance to representative European waste-management policies with quantifiable targets. The usefulness of the WDI for each quantitative policy target is qualitatively rated as either ‘weak’ or ‘strong’.

## Supplementary information

Supplementary Information

## Source data

Source Data

## Data Availability

Data supporting the findings of this study are derived from the Waste Data Interrogator. Processed datasets generated during the analysis are available in the Source Data file provided with this paper.
