# Supplementary material for: Collection and reporting of waste data to support waste management policies
Source: Npj Mater Sustain. 2026 Feb 21;4(1):6. doi: 10.1038/s44296-025-00092-6 (PMC12924763; doi:10.1038/s44296-025-00092-6)
Supplement: Supplementary file 1 — Supplementary Information [file 44296_2025_92_MOESM1_ESM.pdf]

Collection and Reporting of Waste Data to Support Waste Management Policies: Supporting Information

Chunbo Zhang, Léonel Tchadjié Noumbissié, Jishuo Zhang, Chi Zhang, Stijn van Ewijk, Julia A. Stegemann*

Department of Civil, Environmental and Geomatic Engineering, University College London, London, WC1E 6BT, UK

Correspondence: j.stegemann@ucl.ac.uk

Contents

1. Supplementary Table

2. Supplementary Figure

2.1 Trends of CD&E waste received in England

2.2 Waste management and recovery rate

2.3 Reliability check

References

1. Supplementary Table

Supplementary Table 1. Recovery and Disposal (R&D) codes. Source: EU Waste Framework Directive[1]

Code	Explanation
R1	Use principally as a fuel or other means to generate energy
R2	Solvent reclamation/regeneration
R3	Recycling/reclamation of organic substances which are not used as solvents (including composting and other biological transformation processes)
R4	Recycling/reclamation of metals and metal compounds
R5	Recycling/reclamation of other inorganic materials
R6	Regeneration of acids or bases
R7	Recovery of components used for pollution abatement
R8	Recovery of components from catalysts
R9	Oil re-refining or other reuses of oil
R10	Land treatment resulting in benefit to agriculture or ecological improvement
R11	Use of waste obtained from any of the operations numbered R1 to R10
R12	Exchange of waste for submission to any of the operations numbered R1 to R11
R13	Storage of waste pending any of the operations numbered R1 to R12 (excluding temporary storage, pending collection, on the site where the waste is produced)
D1	Deposit into or on to land (e.g. landfill, etc.)
D2	Land treatment (e.g. biodegradation of liquid or sludgy discards in soils, etc.)
D3	Deep injection (e.g. injection of pumpable discards into wells, salt domes or naturally occurring repositories, etc.)
D4	Surface impoundment (e.g. placement of liquid or sludgy discards into pits, ponds or lagoons, etc.)
D5	Specially engineered landfill (e.g. placement into lined discrete cells which are capped and isolated from one another and the environment, etc.)
D6	Release into a water body except seas/oceans
D7	Release to seas/oceans including sea-bed insertion
D8	Biological treatment not specified elsewhere in this Annex which results in final compounds or mixtures which are discarded by means of any of the operations numbered D1 to D12
D9	Physico-chemical treatment not specified elsewhere in this Annex which results in final compounds or mixtures which are discarded by means of any of the operations numbered D1 to D12 (e.g. evaporation, drying, calcination, etc.)
D10	Incineration on land
D11	Incineration at sea
D12	Permanent storage (e.g. emplacement of containers in a mine, etc.)
D13	Blending or mixing prior to submission to any of the operations numbered D1 to D12
D14	Repackaging prior to submission to any of the operations numbered D1 to D13
D15	Storage pending any of the operations numbered D1 to D14 (excluding temporary storage, pending collection, on the site where the waste is produced)

Supplementary Table 2. Summary of typical Statistical Quality criteria

Source	Statistical Evaluation Framework
Eurostat[2]	**1. Relevance:** the degree to which statistics meet current and potential needs of the users<br>**2. Accuracy:** the closeness of estimates to the unknown true values<br>**3. Timeliness:** the period between the availability of the information and the event or phenomenon it describes<br>**4. Punctuality:** the delay between the date of the release of the data and the target date (the date by which the data should have been delivered)<br>**5. Accessibility and Clarity:** the conditions and modalities by which users can obtain, use and interpret data<br>**6. Comparability:** the measurement of the impact of differences in applied statistical concepts, measurement tools and procedures where statistics are compared between geographical areas, sectoral domains or over time<br>**7. Coherence:** the adequacy of the data to be reliably combined in different ways and for various uses
OECD[3]	**1. Relevance:** the degree to which the data serves to address the purposes for which they are sought by users<br>**2. Accuracy:** the degree to which the data correctly estimate or describe the quantities or characteristics they are designed to measure<br>**3. Credibility:** the confidence that users place in those products based simply on their image of the data producer, i.e., the brand image<br>**4. Timeliness:** the length of time between their availability and the event or phenomenon they describe, but considered in the context of the time period that permits the information to be of value and still acted upon<br>**5. Accessibility:** how readily the data can be located and accessed from within OECD data holdings<br>**6. Interpretability:** the ease with which the user may understand and properly use and analyse the data<br>**7. Coherence:** the degree to which they are logically connected and mutually consistent<br>**8. Cost-efficiency:** the costs and provider burden relative to the output.
Statistics Canada[4]	**1. Relevance**: the degree to which statistical information meets user needs<br>**2. Accuracy**: the degree to which statistical information correctly describes the phenomena it was designed to measure<br>**3. Timeliness**: the delay between the end of the reference period to which statistical information pertains and the date on which the information becomes available<br>**4. Accessibility**: the ease with which statistical information can be obtained<br>**5. Coherence:** the degree to which statistical information is logically consistent and can be brought together with information from other sources or different time periods<br>**6. Interpretability:** the availability of supplementary information (metadata) necessary to understand, analyse and utilise statistical information appropriately
Statistics Sweden[5]	**1. Contents:** statistical target characteristics; comprehensiveness<br>**2. Accuracy:** overall accuracy; sources of inaccuracy; presentation of accuracy measures<br>**3. Timeliness:** frequency; production time; punctuality<br>**4. Coherence especially Comparability:** comparability over time; comparability over space; coherence in general<br>**5. Availability and Clarity:** forms of dissemination; presentation; documentation; access to micro data; information services
UK Statistics Authority[6]	**1. Trustworthiness:** confidence in the people and organisations that produce statistics and data, including T1 Honesty and integrity, T2 Independent decision making and leadership, T3 Orderly release, T4 Transparent processes and management, T5 Professional capability, T6 Data governance<br>**2. Quality:** Data and methods that produce assured statistics, including Q1 Suitable data sources; Q2 Sound methods; Q3 Assured quality<br>**3. Value:** statistics that support society's needs for information, including V1 Relevance to users, V2 Accessibility, V3 Clarity and insight, V4 Innovation and improvement, V5 Efficiency and proportionality

Supplementary Table 3. Criteria used in this study and their relevance to other criteria

Criteria of this study	Criteria involved
Effectiveness of classification	**Eurostat:** 1. Relevance, 2. Accuracy, 3. Timeliness, 4. Punctuality<br>**OECD:** 1. Relevance, 2. Accuracy, 4. Timeliness, 6. Interpretability<br>**Statistics Canada:** 1. Relevance, 2. Accuracy, 3. Timeliness, 6. Interpretability<br>**Statistics Sweden:** 1. Contents, 2. Accuracy, 3. Timeliness,<br>**UK Statistics Authority:** 1. Trustworthiness, 3. Value
Comprehensiveness of waste information	**Eurostat:** 1. Relevance, 3. Timeliness, 5. Accessibility and Clarity<br>**OECD:** 1. Relevance, 4. Timeliness, 6. Interpretability,<br>**Statistics Canada:** 1. Relevance, 3. Timeliness, 6. Interpretability<br>**Statistics Sweden:** 1. Contents, 3. Timeliness, 5. Availability and Clarity<br>**UK Statistics Authority:** 3. Value
Suitability for spatial analysis	**Eurostat:** 1. Relevance<br>**OECD:** 1. Relevance<br>**Statistics Canada:** 1. Relevance<br>**Statistics Sweden:** 1. Contents<br>**UK Statistics Authority:** 3. Value
Clarity and user-friendliness	**Eurostat:** 5. Accessibility and Clarity<br>**OECD:** 5. Accessibility, 6. Interpretability<br>**Statistics Canada:** 4. Accessibility, 6. Interpretability<br>**Statistics Sweden:** 5. Availability and Clarity<br>**UK Statistics Authority:** 3. Value
Data reliability	**Eurostat:** 2. Accuracy, 6. Comparability,7. Coherence:<br>**OECD:** 2. Accuracy, 6. Interpretability, 7. Coherence<br>**Statistics Canada:** 2. Accuracy, 5. Coherence, 6. Interpretability<br>**Statistics Sweden:** 2. Accurary, 4. Coherence especially Comparability<br>**UK Statistics Authority:** 1. Trustworthiness, 2. Quality

2. Supplementary Figure

2.1 Trends of CD&E waste received in England

Historic time-series waste data enable the analysis of trends and patterns in waste generation, recovery rates, disposal methods, and material composition over time. Such analyses help identify long-term changes, emerging issues, and potential future developments in waste management practices. Regarding timeliness and punctuality, the WDI has released waste data annually since 2006, in accordance with the requirements of the Waste (England and Wales) Regulations[31], typically with a two-year reporting delay. As of October 2025, the most recent WDI release corresponds to the year 2023, which is not included in the present analysis. Therefore, the temporal scope spans from 2006 to 2022.

Supplementary Figure 1 illustrates the historical trends for received waste categories and waste receipt regions in England from 2006 to 2022. The absence of Recovery & Disposal (R&D) codes in the datasets before 2017 prevented the exclusion of double-counting for the period from 2006 to 2016. Other research focusing on this period[7,8] may have produced incorrectly higher estimates of waste mass. To overcome this data inconsistency, data from 2006 to 2016 were scaled using a factor of 0.85. This factor was calculated based on the average share (fluctuating between 82.8–85.9%) of construction, demolition, and excavation (CD&E) waste excluding double-counting compared to the total CD&E waste received between 2017 and 2022. Supplementary Figure 1 shows that despite a slight decrease in 2009, 2018, and 2020, the CD&E waste received in England rose from 33 Mt to 76 Mt during the 17-year survey period, mainly due to an increase in excavation waste.

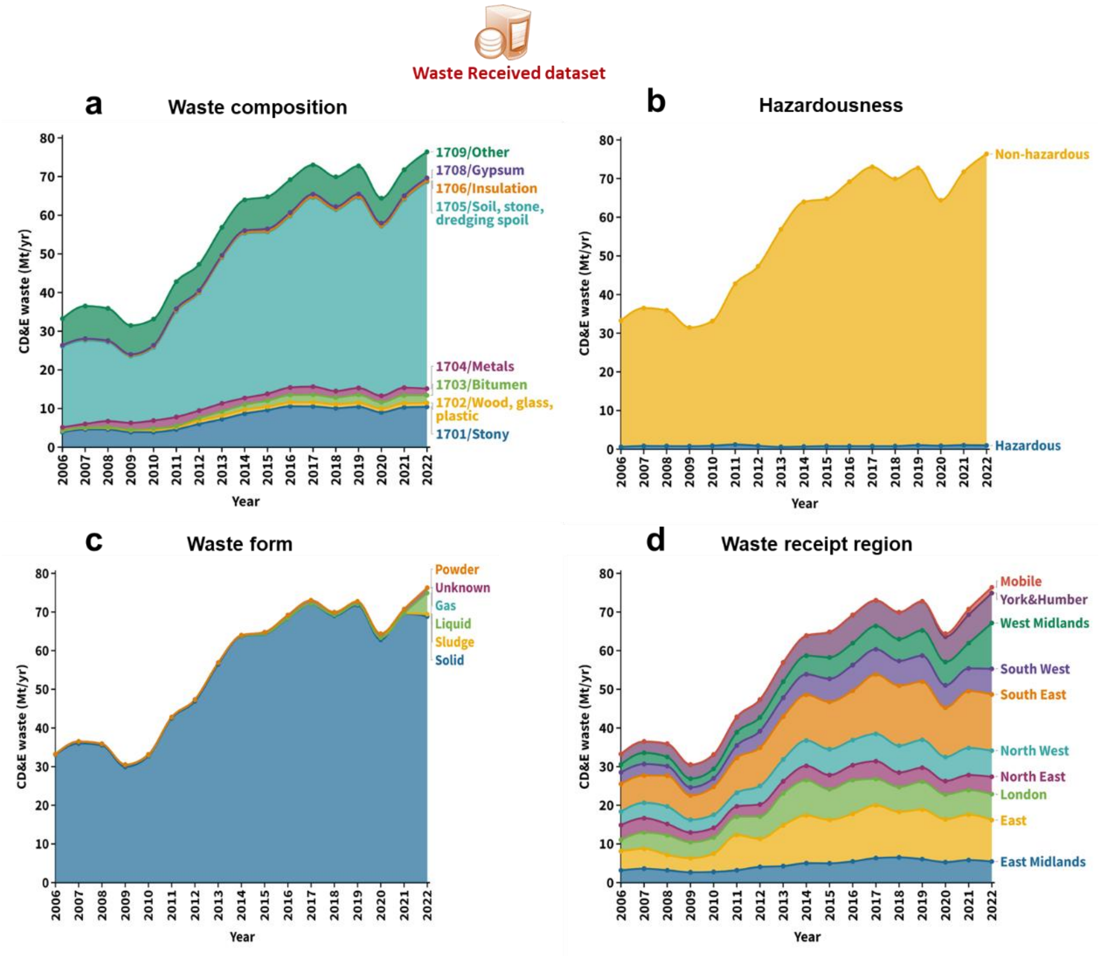

Supplementary Figure 1. 72 Mt of Construction, demolition, and excavation (CD&E) waste received in England for the peroid of 2006–2022. (a) Breakdown based on waste composition. (b) Breakdown based on waste hazardousness (c) Breakdown based on waste form, (d) Breakdown based on the amount of CD&E waste received in each region of England. Note: the trend of waste receipt based on the EWC-stat breakdown is not available, as the EWC-stat codes were used in the WDI since 2020.

2.2 Waste management and recovery rate

Supplementary Figure 2 shows the CD&E waste management in England in 2021 from Origin regions to final fate.

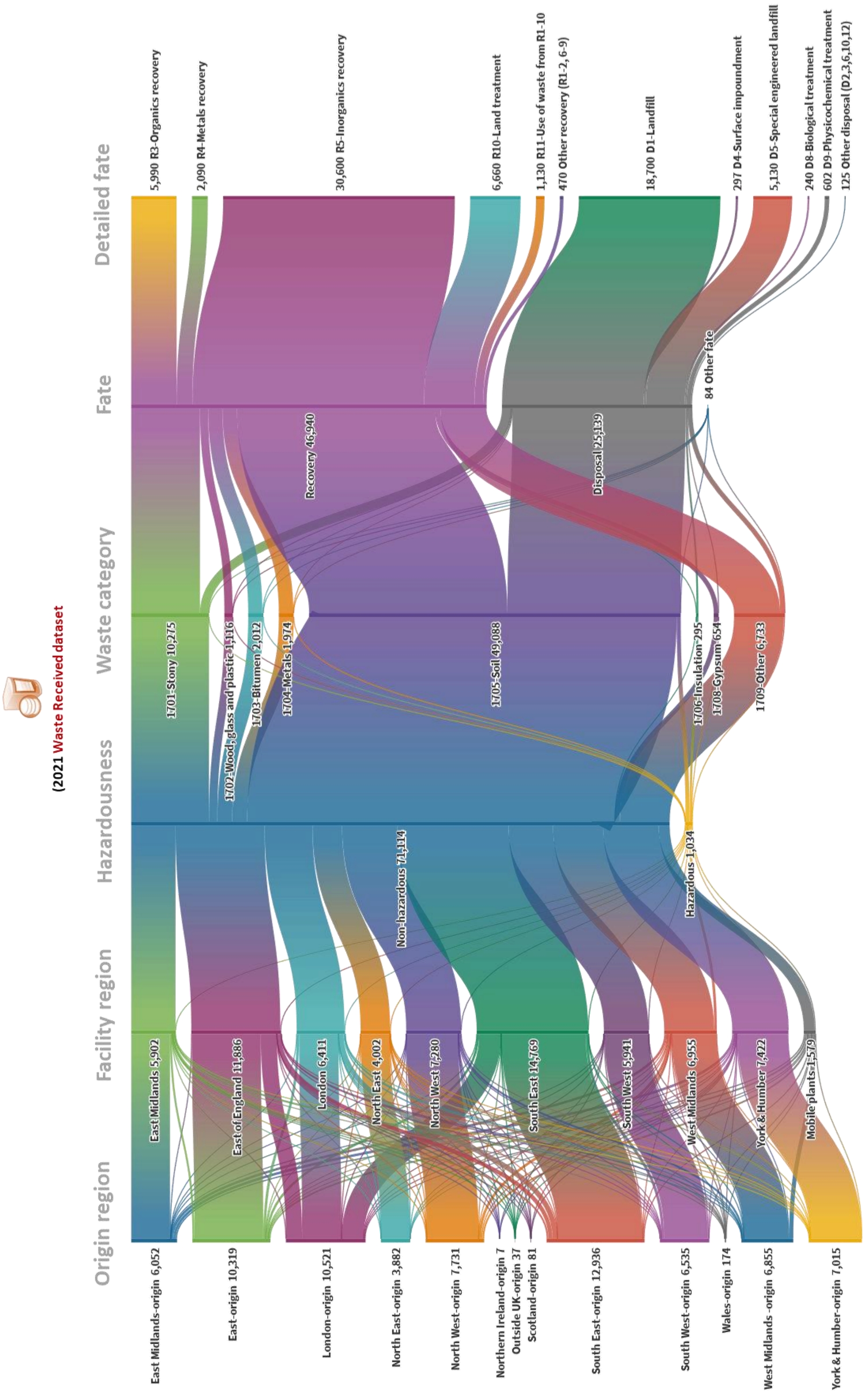

Supplementary Figure 2. Sankey diagram of 72 Mt of construction, demolition, and excavation (CD&E) waste from Waste Received dataset in England in 2021 (Unit: Kilotonne (Kt)).

As the R&D codes were not disclosed in the datasets from 2006 to 2016, Supplementary Figure 3 can show only the fate of CD&E waste treated from 2017 to 2022. Most CD&E waste has been recovered, with the recovery rate continuously increasing from 53–64%. However, there was still around 25 Mt of CD&E waste disposed of per year.

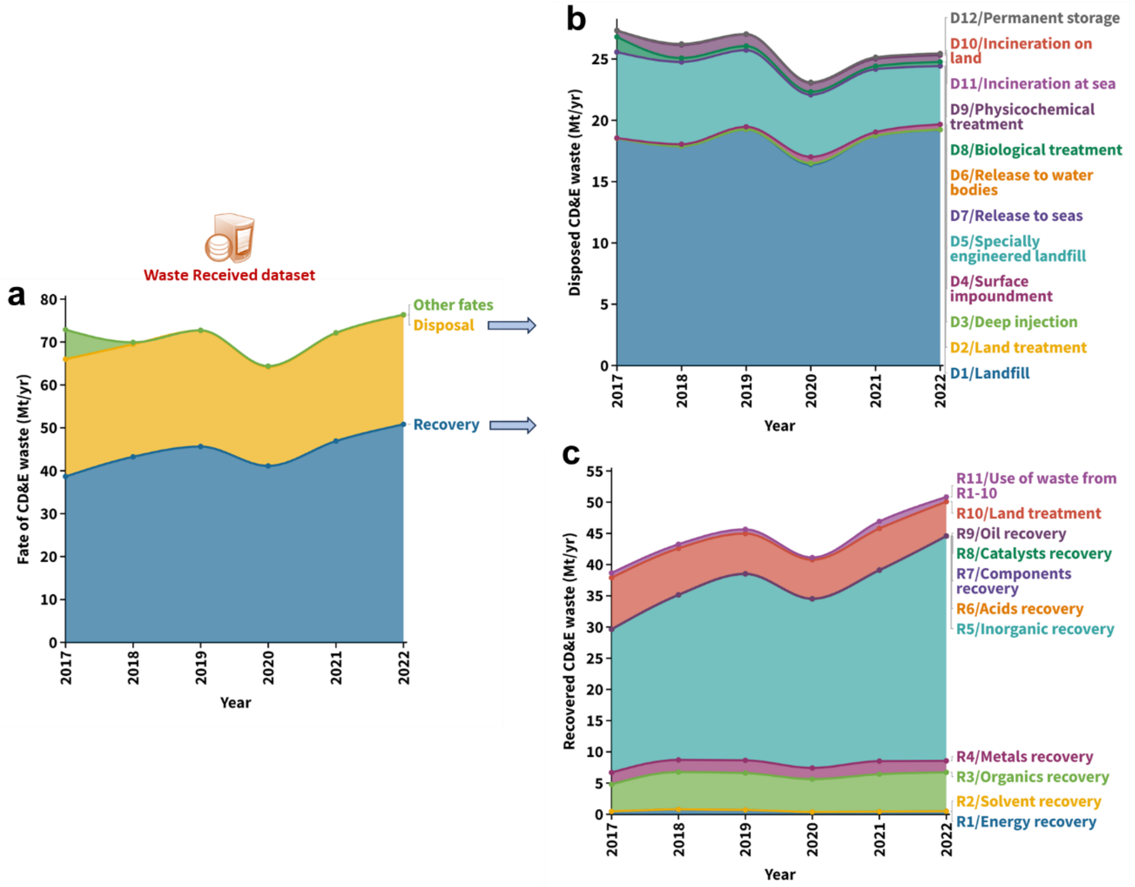

Supplementary Figure 3. Fate of construction, demolition, and excavation (CD&E) waste received in England from 2017 to 2022 based on the Recovery and Disposal (R&D) codes[1]. (a) Breakdown of CD&E waste treatment based on recovery, disposal, and other fates. (b) Detailed disposal routes. (c) Detailed recovery routes.

The waste recovery rate is a widely used indicator to evaluate waste management and assess progress toward the Zero Avoidable Waste (ZAW) target. The recovery rates of overall CD&E waste and its component waste streams in 2021 are shown in Supplementary Figure 4. Notably, the treatment of hazardous wastes to eliminate their hazardousness results in a similar recovery rate (56%) to that of non-hazardous waste (65%). Compared to recovery rates of over 90% for most CD&E wastes, that of insulation wastes (17 06) was poor, at 17%, especially for asbestos (17 06 01, 0.1%). Soil, stone, and dredging spoil (17 05) also had relatively low recovery rates of 53%. The data collected in the WDI thus reveal that the large quantities of unrecovered insulation and excavation wastes are major obstacles to achieving the ZAW goal in the construction sector, despite a high potential for soil recovery[9]. Estimates suggest that implementing measures such as increased landfill taxation, legislation to facilitate reuse, and promoting intermediate storage could reduce soil landfill disposal to 75% of the 2020 level by 2040 and achieve complete elimination by 2050[10]. If all non-hazardous soil were reclaimed, the overall CD&E waste recovery rate could rise from the current 64% to an impressive 95%.

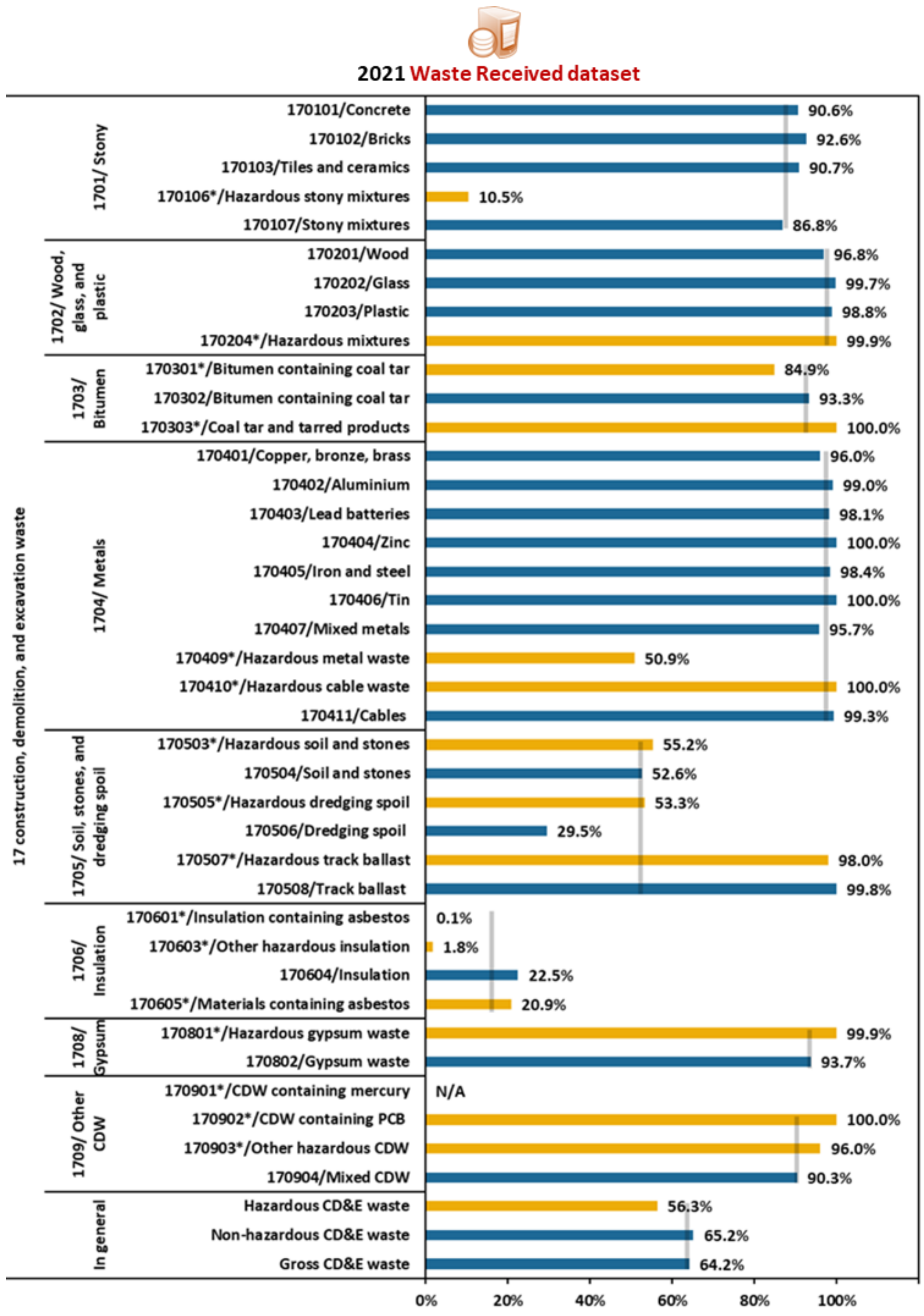

Supplementary Figure 4. Recovery rates of 72 Mt of construction, demolition, and excavation (CD&E) wastes, by European List of Wastes (LoW) code based on the Waste Received dataset in England in 2021. Note: The recovery rate of each waste stream is calculated as the amount of waste coded under the R category of the Recovery & Disposal (R&D) codes relative to the total amount of waste received. Landfilled CD&E waste recorded under code 19 12 12 is excluded.

Ballast (17 05 07/08) was almost entirely recovered, regardless of whether it is hazardous or not. Due to their high economic value, metal wastes (17 04) have the highest recovery rate of approximately 99%, followed by wood, glass, and plastic, with 98% (17 02). Separation makes it easier to process and recycle waste. Although the mixed waste stream (17 09 04) has a recovery rate of over 90%, enhancing the separation of mixed waste can enable higher-value recycling and provide associated data. It is noted that a portion of CD&E waste sent to landfill is recorded under Chapter 19 (see Section 2.2), which may lead to overestimation of recovery rates. For example, while the recovery rate of gypsum was 94% in England in 2021, it was only 13% in Europe in 2013[11]. It is also

important to address that the existing recovery codes are too broad to distinguish between recovery options in the waste hierarchy, limiting the ability to assess the material efficiency in recovery.

The WDI also enables the investigation of regional differences in waste treatment. Supplementary Figure 5 compares the recovery rates, local treatment rates, and mobile treatment rates of the nine regions in England. London has a noticeably higher recovery rate at 95%, compared with other regions (53–75%). Most CD&E waste was treated in its region of origin, which reduces costs and impacts of its transport. Although mobile recovery can significantly reduce waste transport[12], it is not yet widely implemented in England, with the highest mobile treatment rate in the West Midlands region at 8.5%. Because the WDI gathers waste data from each facility and then aggregates it for each region in England, treatment can also be broken down from the country level to the site level.

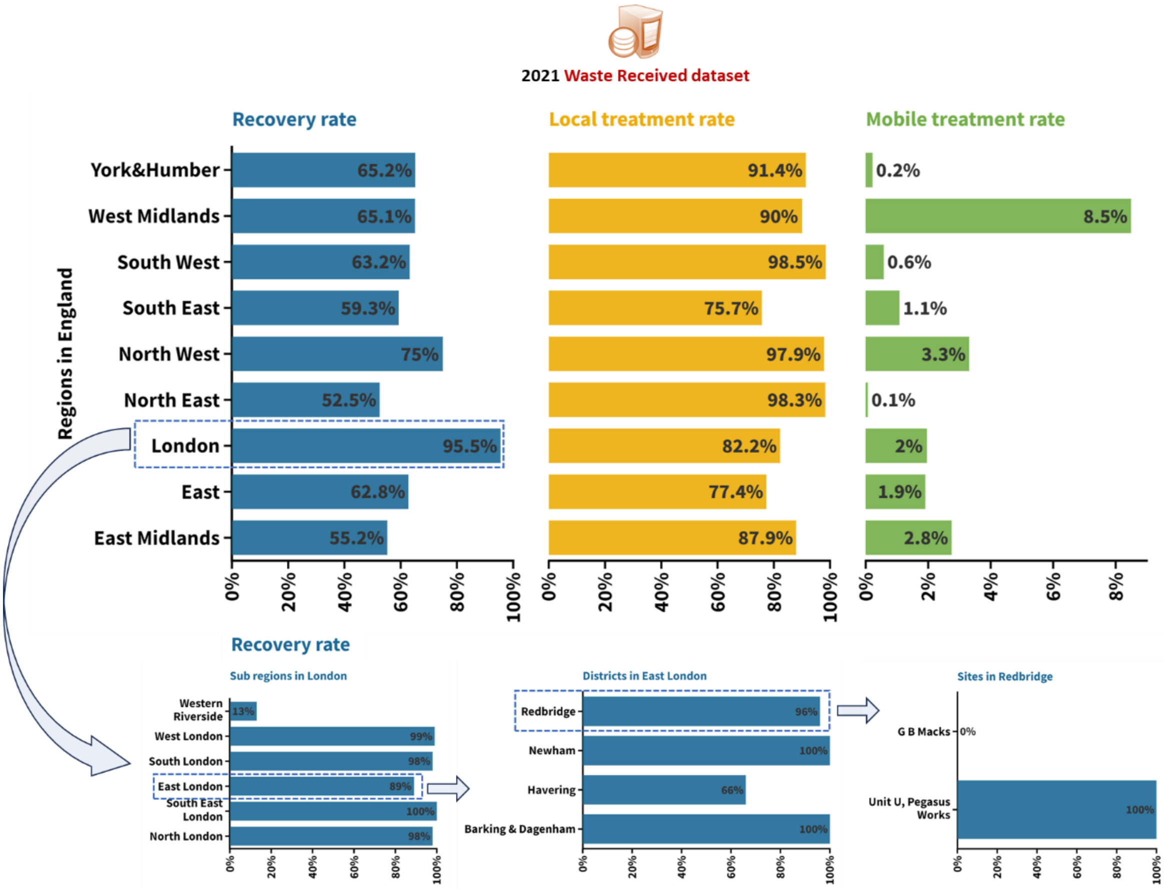

Supplementary Figure 5. Comparison of recovery rates and treatment patterns of 72 Mt of construction, demolition, and excavation (CD&E) waste at different scales based on the 2021 Waste Received dataset. Note: local treatment rate is the proportion of waste treated in its origin region; mobile treatment rate is the proportion of waste treated through mobile plants.

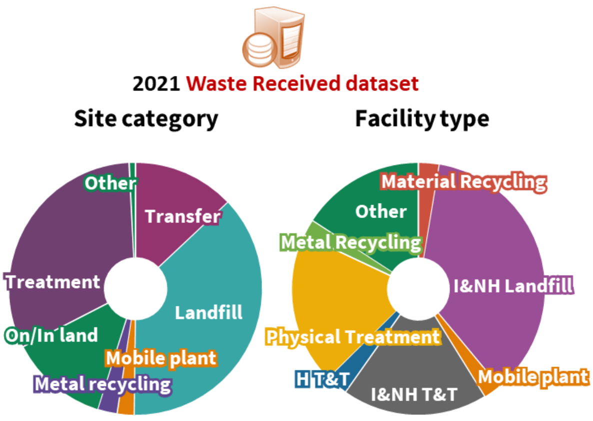

Supplementary Figure 6. Breakdown of 72 Mt of construction, demolition, and excavation (CD&E) waste based on site category and facility type using the 2021 Waste Received dataset. Note: HT&T: Hazardous waste transfer and treatment, I&NH T&T: Inert and non-hazardous waste transfer and treatment.

2.3 Reliability check

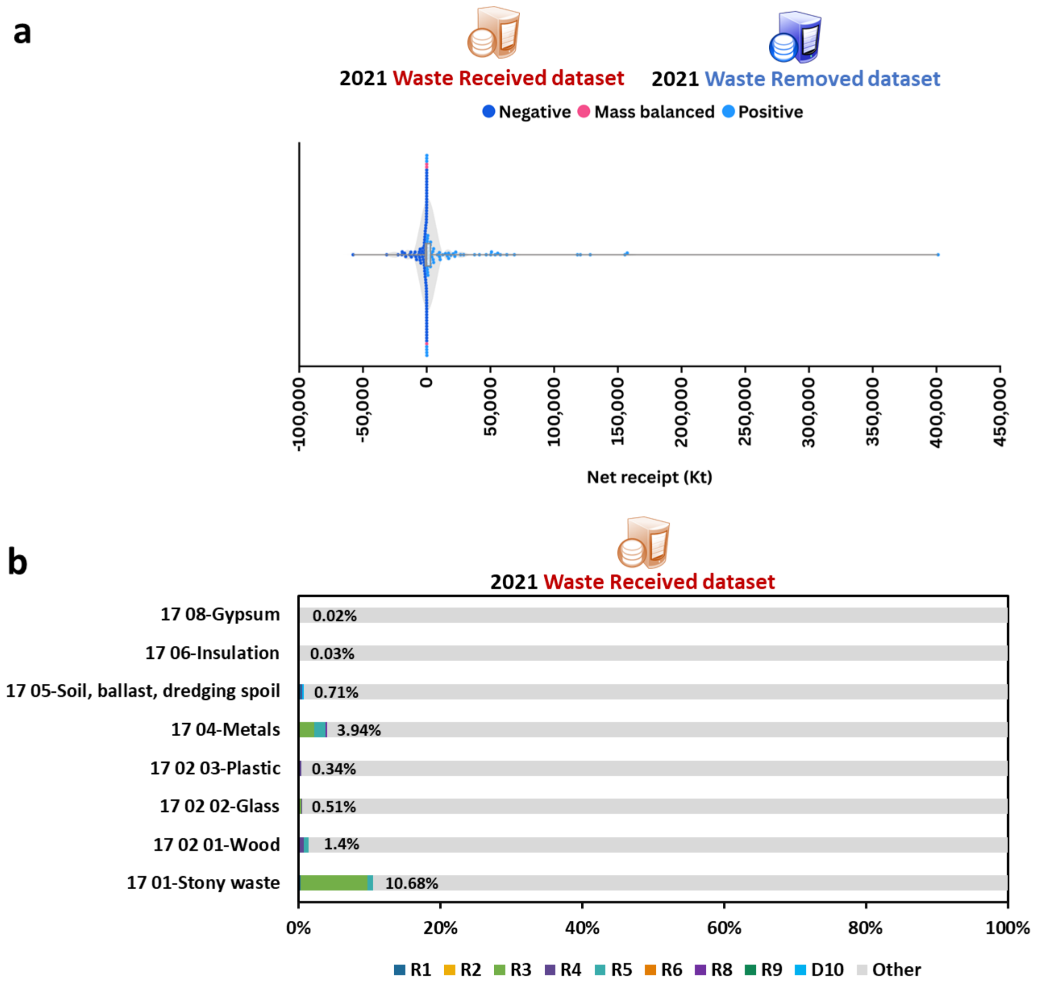

Supplementary Figure 7. Reliability check. (a) Waste received minus waste removed for all 151 sites in Greater Manchester in 2021. (b) Share of improbable fates of each waste stream compared to the total waste treatment in the England.

References

1. EC. Directive 2008/98/EC of the European Parliament and of the Council of 19 November 2008 on waste and repealing certain Directives (Text with EEA relevance). https://eur-lex.europa.eu/legal-content/EN/TXT/?uri=CELEX:32008L0098 (2008).

2. Eurostat. *European Statistical System handbook for quality and metadata reports*. https://ec.europa.eu/eurostat/web/products-manuals-and-guidelines/-/ks-gq-19-006 (2020) doi:10.2785/666412.

3. OECD. *Quality Framework and Guidelines for OECD Statistial Activities*. https://www.oecd.org/sdd/qualityframeworkforoecdstatisticalactivities.htm (2012).

4. Statistics Canada. *Statistics Canada's Quality Assurance Framework - Third Edition*. https://www150.statcan.gc.ca/n1/pub/12-586-x/12-586-x2017001-eng.pdf (2017).

5. Statistics Sweden. *Quality Concept for Official Statistics Statistics*. https://www.scb.se/contentassets/14f5e346f4814dd0acd52d10b23286c6/rnd-report-1997-02-green.pdf (1997).

6. UK Statistics Authority. *Code of Practice for Statistics: Ensuring official statistics serve the public*. https://code.statisticsauthority.gov.uk/ (2022).

7. Grieken, J. Van. Scenario-based system assessment for business strategy development in the Circular Economy – A case study of Enviro Waste London Ltd. (2017).

8. Tomlinson, S. J., E.J.Carnell, Tang, Y. S., Sutton, M. A. & Dragosits, U. *Ammonia emissions from UK non-agricultural sources in 2016: Contribution to the National Atmospheric Emission Inventory*. https://nora.nerc.ac.uk/id/eprint/518940/1/N518940CR.pdf (2017).

9. Kourmouli, A. *et al. Potential for a Soil Reuse and Storage system in England*. (2024) doi:10.5281/zenodo.10943857.

10. CLC. The Routemap for zero avoidable waste in construction. (2021).

11. Jiménez Rivero, A., Sathre, R. & García Navarro, J. Life cycle energy and material flow implications of gypsum plasterboard recycling in the European Union. *Resour. Conserv. Recycl.* **108**, 171–181 (2016).

12. Zhang, C. *et al.* Eco-efficiency assessment of technological innovations in high-grade concrete recycling. *Resour. Conserv. Recycl.* **149**, 649–663 (2019).
